# Structure–function analysis of HsiF, a gp25-like component of the type VI secretion system, in *Pseudomonas aeruginosa*

**DOI:** 10.1099/mic.0.051987-0

**Published:** 2011-12

**Authors:** Nadine S. Lossi, Rana Dajani, Paul Freemont, Alain Filloux

**Affiliations:** 1 Centre for Molecular Microbiology and Infection (CMMI), Division of Cell and Molecular Biology, Imperial College London, London SW7 2AZ, UK; 2 Division of Molecular Biosciences, Imperial College London, London SW7 2AZ, UK

## Abstract

Bacterial pathogens use a range of protein secretion systems to colonize their host. One recent addition to this arsenal is the type VI secretion system (T6SS), which is found in many Gram-negative bacteria. The T6SS involves 12–15 components, including a ClpV-like AAA^+^ ATPase. Moreover, the VgrG and Hcp components have been proposed to form a puncturing device, based on structural similarity to the tail spike components gp5/gp27 and the tail tube component gp19 of the T4 bacteriophage, respectively. Another T6SS component shows similarity to a T4 phage protein, namely gp25. The gp25 protein has been proposed to have lysozyme activity. Other T6SS components do not exhibit obvious similarity to characterized T4 phage components. The genome of *Pseudomonas aeruginosa* contains three T6SS gene clusters. In each cluster a gene encoding a putative member of the gp25-like protein family was identified, which we called HsiF. We confirmed this similarity by analysing the structure of the *P. aeruginosa* HsiF proteins using secondary and tertiary structure prediction tools. We demonstrated that HsiF1 is crucial for the T6SS-dependent secretion of Hcp and VgrG. Importantly, lysozyme activity of HsiF proteins was not detectable, and we related this observation to the demonstration that HsiF1 localizes to the cytoplasm of *P. aeruginosa*. Finally, our data showed that a conserved glutamate, predicted to be required for proper HsiF folding, is essential for its function. In conclusion, our data confirm the central role of HsiF in the T6SS mechanism, provide information on the predicted HsiF structure, and call for reconsideration of the function of gp25-like proteins.

## Introduction

Pathogenic bacteria adopt different lifestyles while infecting their host (Merrell & Falkow, 2004). A broad arsenal of virulence factors ensures bacterial survival and successful establishment of acute or chronic infections. Protein secretion systems are the weapons of choice in the course of an infection (Bleves *et al.*, 2010). In many cases, including that of the opportunistic pathogen *Pseudomonas aeruginosa*, the type III secretion system (T3SS) has been linked to increased mortality, persistence, relapse, and higher bacterial burdens in acutely infected patients (Hauser *et al.*, 2002; Hauser, 2009; Roy-Burman *et al.*, 2001). Another secretion system has been the subject of intense investigation during the last five years: the type VI secretion system (T6SS). In several studies, *T6SS* genes have been induced upon colonization of the host (Bingle *et al.*, 2008; Cascales, 2008; Filloux *et al.*, 2008). In contrast to the T3SS, the *P. aeruginosa* T6SS has been shown to be associated with chronic infection. It has been shown that sera of cystic fibrosis patients chronically infected with *P. aeruginosa* contain antibodies directed against the T6SS component Hcp, which suggests that the T6SS is highly active in this context (Mougous *et al.*, 2006). Furthermore, high-throughput screening for *P. aeruginosa* mutants attenuated in a rat model of chronic respiratory infection has demonstrated that *T6SS* genes are essential for bacterial survival (Potvin *et al.*, 2003). However, it has recently been proposed that one of the *P. aeruginosa* T6SSs is required to compete with other bacteria, in particular by allowing the release of a set of toxins named Tse1–3 (Hood *et al.*, 2010; Schwarz *et al.*, 2010). In a recent study, it was shown that both Tse1 and Tse3 can be delivered to the periplasm of recipient bacteria, and that these proteins are able to hydrolyse peptidoglycan (Russell *et al.*, 2011).

In *P. aeruginosa*, three *T6SS* gene clusters have been identified, namely *H1-* to *H3-T6SS* (Filloux *et al.*, 2008). Whereas *H1-T6SS* gene expression is controlled by the RetS/LadS signalling pathway (Goodman *et al.*, 2004; Records & Gross, 2010; Ventre *et al.*, 2006), *H2-T6SS* and *H3-T6SS* genes have been proposed to be under control of the quorum-sensing circuitry (Lesic *et al.*, 2009). The number of genes and the genetic organization vary from one cluster to the other (Filloux *et al.*, 2008), but a set of genes encoding T6SS core components has been identified. One of these is the *clpV* gene, which encodes a protein from the AAA^+^ ATPase family. In *Vibrio cholerae*, ClpV has been shown to modulate the assembly of tubular complexes formed by two components of the *V. cholerae* T6SS machinery, namely VipA and VipB (Bönemann *et al.*, 2009, 2010; Filloux, 2009).

Little is known about the function of genes contained in *T6SS* clusters and whether all are needed for T6SS function. In *Edwardsiella tarda*, a systematic study has been performed, in which it has been found that 13 out of the 16 predicted T6SS components (Evp) are required for EvpP secretion (Zheng & Leung, 2007). Furthermore, how the T6SS machinery assembles and promotes transport of proteins across the bacterial cell envelope is also largely unknown. Finally, which proteins are secreted via the T6SS is poorly understood and has been clearly reported only in a few cases, e.g. the three T6SS substrates Tse1–3, recently identified in *P. aeruginosa* (Hood *et al.*, 2010).

In most Gram-negative bacteria carrying a T6SS, two core T6SS components are present as T6SS substrates, namely the VgrG and Hcp proteins (Mougous *et al.*, 2006; Pukatzki *et al.*, 2007). Intriguingly, the most remarkable feature of VgrG and Hcp proteins is their structural resemblance to the tail components of the T4 bacteriophage (Leiman *et al.*, 2009, 2010). VgrG proteins resemble the gp27/gp5 trimeric complex used by the phage to puncture the bacterial cell membrane and initiate DNA injection. The puncturing device sits on top of the phage tail tube used by the phage to inject DNA into bacteria (Leiman *et al.*, 2009). Interestingly, Hcp proteins show structural similarity to gp19, which forms the tail tube. This resemblance is supported by the fact that Hcp proteins form hexameric rings *in vitro*. These structures have a central channel of 40 Å in diameter (Mougous *et al.*, 2006) and assemble into nanotubes (Ballister *et al.*, 2008; Leiman *et al.*, 2009). These observations suggest that the T6SS forms an inverted puncturing device, perforating the bacterial cell envelope from the inside to the outside (Pukatzki *et al.*, 2009). There is therefore a possibility that T6SS substrates are secreted through the Hcp tube or interact directly with VgrG proteins at the tip of the tube. In this way they could be transported through the bacterial membrane concomitantly with the VgrG proteins (Hachani *et al.*, 2011). The latter possibility is based on the observation that some VgrG proteins carry C-terminal domains displaying specific activity, such as actin remodelling activity in VgrG1 from *V. cholerae* (Ma *et al.*, 2009) and *Aeromonas hydrophila* (Suarez *et al.*, 2010). Consequently, these C-terminal extensions are considered effector proteins of the T6SS, though they are covalently linked to one of its components. In *P. aeruginosa*, three VgrG proteins are co-regulated with the *H1-T6SS* cluster, namely VgrG1a (PA0091), VgrG1b (PA0095) and VgrG1c (PA2685) (Hood *et al.*, 2010; Mougous *et al.*, 2006). Whereas VgrG1a and VgrG1c are secreted in an H1-T6SS-dependent manner, VgrG1b is still secreted in a *clpV1* mutant and has been found to be secreted independently of all three *P. aeruginosa* T6SSs (Hachani *et al.*, 2011).

In this study, we have investigated the role of an as-yet-uncharacterized *P. aeruginosa* T6SS component, called HsiF. HsiF is also called TssE (Hood *et al.*, 2010), but we use the Hsi nomenclature as previously described (Filloux *et al.*, 2008), which matches the nomenclature described for the *Rhizobium leguminosarum* T6SS archetype (Bladergroen *et al.*, 2003). We show that HsiF proteins are essential components of the T6SS, and we further support the hypothesis that this family of proteins is structurally similar to the T4 phage protein gp25. Furthermore, our data suggest that HsiF proteins do not display lysozyme activity, as has been suggested for members of the gp25-like protein family (Szewczyk *et al.*, 1986). In agreement with this observation, we demonstrate that HsiF is localized in the *P. aeruginosa* cytoplasm.

## Methods

### 

#### Bacterial strains and growth conditions.

Bacterial strains used in this study are described in Supplementary Table S1. *P. aeruginosa* strains were grown in tryptone soy broth (TSB; Difco) supplemented with antibiotics as appropriate (carbenicillin, 150 µg µl^−1^; streptomycin, 2000 µg µl^−1^). *Escherichia coli* strains were grown in Luria–Bertani (LB; Difco) broth supplemented with antibiotics as appropriate (kanamycin, 50 µg µl^−1^; ampicillin, 100 µg µl^−1^; streptomycin, 50 µg µl^−1^).

#### Antibodies and reagents.

Polyclonal anti-VgrG1a, anti-VgrG1b and anti-Hcp1 have been described previously (Hachani *et al.*, 2011) and were used at a dilution of 1 : 1000. Monoclonal anti-RNA polymerase (RNAP) antibody (W0023, Neoclone), directed against the beta subunit of RNAP, was used at a dilution of 1 : 1000. Polyclonal anti-HsiF1 was used at a dilution of 1 : 500 and obtained as described above. Polyclonal anti-DsbA was used at a dilution of 1 : 10 000 and was kindly provided by Dr Karl E. Jaeger (IMET, Jülich, Germany). Polyclonal anti-XcpY antibody has been described previously (Michel *et al.*, 1998) and was used at a dilution of 1 : 1000. Secondary antibodies were purchased from Sigma and used at a dilution of 1 : 5000 [goat anti-rabbit–horseradish peroxidase (HRP) (A6154); rabbit anti-mouse–HRP (A 9044)]. Western blots were developed using SuperSignal West Pico Chemiluminescent Substrate (Pierce) and a Fuji LAS-3000 imager. PCRs for cloning purposes were carried out using High Fidelity DNA polymerase (Roche).

#### Plasmids.

Plasmids used in this study are summarized in Supplementary Table S2. The expression plasmids pET-F1, encoding an N-terminal 6×His fusion protein of HsiF1, and pGEX-F3, encoding an N-terminal glutathione *S*-transferase (GST) fusion of HsiF3, were constructed as follows. *hsiF1* and *hsiF3* were amplified from genomic DNA of *P. aeruginosa* PAO1 by PCR using oligonucleotide pairs OAL92/94 and OAL99/100 (Supplementary Table S3), respectively, adding appropriate restriction sites to the respective ends of the PCR product. Inserts were confirmed by sequencing and inserted into pET28a (Stratagene) and pGEX4T1 (GE Healthcare), respectively. The complementation plasmid pHsiF1 was constructed by amplification of *hsiF1* from *P. aeruginosa* PAK genomic DNA using OAL138/139, adding *Eco*RI and *Bam*HI restriction sites to the respective ends of the PCR product for ligation into pBBR1MCS-4. The *hsiF1_E105A_* allele was engineered by site-directed mutagenesis using the QuikChange Site-Directed Mutagenesis kit (Stratagene) according to the manufacturer’s manual, employing primers OAL147/OAL148 containing the desired mutation. The plasmid pKNG-Δ*hsiF1* was constructed as follows. Overlapping gene segments were amplified from *P. aeruginosa* PAK genomic DNA upstream and downstream of *hsiF1* using oligonucleotide pairs OAL65/66 and OAL67/68, and were merged by overlapping PCR using primers OAL65/68. The resulting DNA fragment was cloned into pKNG101 using *Apa*I and *Bam*HI restriction sites. All constructs were confirmed by sequencing (GATC Biotech) prior to use.

#### Construction of deletion mutants in *P. aeruginosa.*

*P. aeruginosa* deletion mutants were constructed as described previously (Vasseur *et al.*, 2005) using the suicide plasmid pKNG101 (Kaniga *et al.*, 1991). To create the PAKΔ*retS*Δ*hsiF1* mutant strain, pKNG-Δ*hsiF1* was introduced into *P. aeruginosa* PAKΔ*retS*. Clones in which the double recombination events occurred, resulting in the deletion of *hsiF1*, were selected on sucrose-containing plates as described elsewhere (Kaniga *et al.*, 1991). Deletion of *hsiF1* was verified by PCR using primers upstream and downstream of *hsiF1* (OAL87/88).

#### Production of antibody directed against HsiF1.

Expression of the 6×His–HsiF1 fusion protein was induced from pET-F1 using 1 mM IPTG (Sigma) in *E. coli* Rosetta2 BL21(DE3) for 6 h at 37 °C before cells were pelleted by centrifugation. Inclusion bodies containing the recombinant 6×His–HsiF1 were isolated from bacterial pellets by sonication on ice in lysis buffer (10 mM Tris, pH 8.0, 3 mM EDTA) and subsequent centrifugation. Inclusion bodies were washed with lysis buffer and dissolved in 8 M urea, pH 8.0, for 1 h at room temperature. Cell debris was removed by ultracentrifugation at 70 000 ***g*** for 1 h at 4 °C, and supernatants were applied to nickel-nitrilotriacetic acid (Ni-NTA) columns (HiTrap, Amersham Biosciences) for purification of His–HsiF1 by imidazole gradient. Eluted 6×His–HsiF1 was dialysed against 10 mM Tris, pH 8.0, and injected into rabbits for antibody production (Eurogentec).

#### HsiF3 production and purification.

Expression of GST fusion protein was induced from pGEX-F3 using 1 mM IPTG (Sigma) in *E. coli* Rosetta2 BL21(DE3) for 16 h at 20 °C before cells were pelleted by centrifugation. Pellets were suspended in lysis buffer [50 mM HEPES, 200 mM NaCl, 5–10 mM DTT, 2 mM EDTA, supplemented with Complete Protease Inhibitor Cocktail (Roche)] and lysed by passage through a French press. The soluble fraction containing GST–HsiF3 was applied to 3 ml Glutathione Sepharose High Performance resin packed into an XK 16/20 column (GE Healthcare), at 0.1 ml min^−1^. The resin was washed with 20 column volumes of lysis buffer and five column volumes of gel filtration buffer (50 mM HEPES, 200 mM NaCl, 2 mM DTT) before overnight incubation at 4 °C with 200 U thrombin to cleave the GST tag. HsiF3 was further purified by gel filtration [applied in gel filtration buffer to a HiLoad 16/60 Superdex 75 column (GE Healthcare)], concentrated using a 4 ml Amicon Ultra Centrifugal filter device (Millipore) and used subsequently in a biochemical assay to analyse its enzymic activity.

#### Preparation of supernatant from *P. aeruginosa* culture.

*P. aeruginosa* strains were grown in TSB overnight, subcultured to OD_600_ 0.1, and grown to early stationary phase at 37 °C under agitation. Cells were separated from culture supernatants by centrifugation of bacterial cultures at 4000 ***g*** at 4 °C. Cells were directly resuspended in 1× Laemmli buffer (Laemmli, 1970). Proteins contained in the culture supernatant were precipitated using 6 M TCA at a final TCA concentration of 10 %. Protein pellets were washed in 90 % acetone, dried, and resuspended in 1× Laemmli buffer for analysis by SDS-PAGE. Samples were boiled at 95 °C for 10 min before SDS-PAGE.

#### Subcellular fractionation of *P. aeruginosa*.

*P. aeruginosa* strains were grown overnight in TSB, and subcultures were grown to early stationary phase the following day. Cells were collected by centrifugation and resuspended in 10 mM Tris, pH 8.0, supplemented with Complete Protease Inhibitor Cocktail (Roche) and 1 mM EDTA, before cells were lysed by sonication (3×30 s, amplitude of 35 %) using a Vibra-Cell ultrasonic processor (Sonics). Intact cells were removed by centrifugation at 4000 ***g*** for 5 min at 4 °C. Soluble and membrane fractions were separated by centrifugation at 100 000 ***g*** for 1 h at 4 °C before analysis by SDS-PAGE.

*Pseudomonas* spheroplasts (cytosol+membranes) and periplasmic fraction were obtained as described elsewhere (Lory *et al.*, 1996). Briefly, cells were collected by centrifugation and washed in 50 mM Tris, pH 7.6. Cell pellets were then suspended in 0.2 M MgCl_2_, 50 mM Tris, pH 7.6, and incubated for 30 min at 30 °C, 5 min on ice and 15 min at room temperature. Spheroplasts were separated from the periplasmic fraction by centrifugation at 8000 ***g*** for 10 min at 4 °C. Samples were suspended in Laemmli buffer and boiled at 95 °C before analysis by SDS-PAGE.

#### Bioinformatic analysis.

Structural prediction and modelling of secondary and tertiary structures were carried out using Phyre (http://www.sbg.bio.ic.ac.uk/phyre) (Kelley & Sternberg, 2009). Tertiary structures were visualized using PyMOL. Amino acid sequence alignments were performed using web-based clustal w2 software (http://www.ebi.ac.uk/Tools/msa/clustalw2) and edited using GenDoc freeware. Phylogenetic analysis was carried out using http://www.phylogeny.fr. The neighbour-joining tree was computed with 100 bootstrap replicates. Tree rendering was carried out using TreeDyn.

#### Assessment of lysozyme activity.

Lysozyme activity of recombinant HsiF3 was assessed using a lysozyme assay described elsewhere (Nakimbugwe *et al.*, 2006). Briefly, 30 µl recombinant HsiF3 (5, 0.5 or 0.05 mg ml^−1^) was incubated with 270 µl *Micrococcus lysodeikticus* suspension [0.35 mg ml^−1^ lyophilized *M. lysodeikticus* (ATCC 4698, Sigma), 0.5 mg BSA ml^−1^ (Sigma) in 10 mM potassium phosphate buffer, pH 7.2]. A 30 µl volume of hen egg white lysozyme (HEWL; 96 000 U mg^−1^, Sigma) was used as a positive control at 0.5 mg ml^−1^, while 30 µl potassium phosphate buffer functioned as a negative control. Cell lysis was monitored as the decrease in turbidity of the enzyme/substrate mixture at 595 nm at 37 °C every 5 min over 1 h using a FLUOstar Optima microplate reader (BMG Labtech). Lysozyme activity was further analysed using an EnzChek Lysozyme Assay kit (E-22013, Molecular Probes) according to the manufacturer’s manual. A 50 µl volume of recombinant HsiF3 (concentration range 5–0.035 mg ml^−1^), HEWL (dilutions as indicated from a stock of 1000 U ml^−1^, provided by the kit), *P. aeruginosa* cell lysate (undiluted) or assay buffer (negative control) was incubated with 50 µl fluorescently labelled *M. lysodeikticus* at 37 °C for 1 h, and fluorescence (excitation 494 nm, emission 518 nm) was measured using a FLUOstar Optima microplate reader at 5 min intervals. *Pseudomonas* cell lysate was prepared from cells grown for 5 h with the appropriate antibiotics in TSB. Cells were pelleted, resuspended in lysis buffer (50 mM Tris, pH 8.0, Complete Protease Inhibitor Cocktail) and lysed by sonication for 4×30 s at an amplitude of 35 % (Vibra-Cell, Sonics). The cell extract was clarified by centrifugation at 16 000 ***g*** for 5 min at 4 °C.

## Results

### HsiF is a conserved T6SS component of the gp25-like protein family

**Fig. 1.**
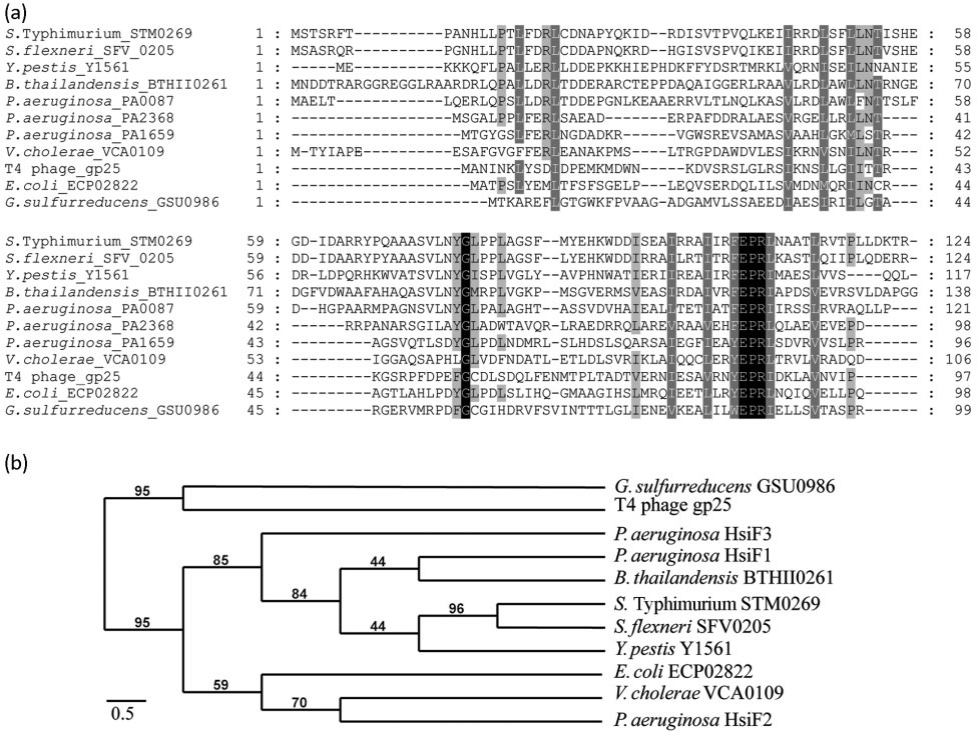
Amino acid sequence comparison and phylogenetic relationship among members of the gp25 family. (a) Identification of conserved residues in members of the gp25-like protein family. Amino acid sequence alignments of HsiF proteins of *P. aeruginosa* (HsiF1/PA0087, HsiF2/PA1659 and HsiF3/PA2368), T4 phage gp25, *G. sulfurreducens* GSU0986 and HsiF homologues found in other Gram-negative bacteria associated with a *T6SS* gene cluster (clustal w2, GeneDoc). Different levels of shading indicate the level of conservation (100 %, black; >80 %, dark grey; >60 %, light grey; <60 %, white). The amino acid position is indicated at the beginning and the end of each line. (b) Phylogenetic analysis of gp25-like proteins. Neighbour-joining tree based on HsiF proteins, gp25 of the T4 bacteriophage, GSU0986 of *G. sulfurreducens* and several HsiF orthologues found in Gram-negative bacteria that are associated with *T6SS* gene clusters. Indicated bootstrap values correspond to 100 replicates. Phylogenetic analysis was carried out using http://www.phylogeny.fr.

The *P. aeruginosa hsiF* gene is one of 12–15 genes conserved in *T6SS* clusters (Filloux *et al.*, 2008). There are three paralogues in *P. aeruginosa*, *hsiF1* (*PA0087*), *hsiF2* (*PA1659*) and *hsiF3* (*PA2368*), which belong to one of the three *T6SS* clusters, *H1-T6SS*, *H2-T6SS* and *H3-T6SS*, respectively. The three *P. aeruginosa* HsiF proteins range from 135 to 169 aa in size, and display from 22.9 % (HsiF1 and HsiF3) to 29.9 % (HsiF2 and HsiF3) sequence identity (Fig. 1a). We compared the *P. aeruginosa* HsiF amino acid sequences with several orthologues found in T6SSs from other bacteria (TIGR03357) (Fig. 1a), including *Salmonella enterica* serovar Typhimurium (*S.* Typhimurium), *Shigella flexneri*, *Yersinia pestis*, *Burkholderia thailandensis*, *V. cholerae* and *E. coli*. The alignment demonstrated that two motifs, EPRL and YGL, are highly conserved among this family of T6SS components, suggesting that they have an essential role.

Furthermore, NCBI blast analysis identified a similarity between HsiF proteins and proteins of the gp25-like family (pfam04965) (Fig. 1a), including the archetype gp25 component of the baseplate wedge of the T4 bacteriophage (Yap *et al.*, 2010), and the gp25-like protein encoded in a prophage region of the *Geobacter sulfurreducens* genome (GSU0986). The three HsiF proteins show similarity to gp25 at the amino acid sequence level, ranging from 16 % (HsiF1 and gp25 from T4 phage) to 21.4 % (HsiF2 and gp25 from T4 phage) identity. The amino acid sequence alignment of the HsiF1, HsiF2, HsiF3 and gp25-like proteins (Fig. 1a) shows a number of highly conserved residues. In particular, the EPRL motif is conserved between HsiFs and gp25-like proteins, while the YGL motif is not conserved among all members of this family (Fig. 1a). The YGL motif is absent both in the T4 phage gp25 and in the gp25-like protein encoded in a prophage region of the *G. sulfurreducens* genome (Fig. 1a). In these proteins, YGL is replaced by an FGC motif, which could be a hallmark of this subfamily. These observations confirmed that the EPRL motif is conserved in all gp25-like proteins, whereas the YGL motif is a hallmark of gp25-like components involved in T6SS.

Phylogenetic analysis confirmed that T6SS members of the gp25 family and the phage-associated gp25 proteins have a common ancestor, but are distinct and found in separate branches of the tree (Fig. 1b). Furthermore, the phylogenetic analysis illustrates that the HsiF proteins of *P. aeruginosa* are not closely related to each other. Instead, each *P. aeruginosa* HsiF is found to be closely related to a homologue from another Gram-negative bacterium (Fig. 1b). For example, HsiF2 shows only 25.5 % identity to HsiF1, but 33.8 % identity to VCA0109 of *V. cholerae*, and HsiF1 displays 22.9 % identity to HsiF3, but 45.8 % identity to BTH_II0261 of *B. thailandensis*. This observation also suggests that each *P. aeruginosa* T6SS may have a distinct function.

### Role of HsiF in VgrG and Hcp secretion

**Fig. 2.**
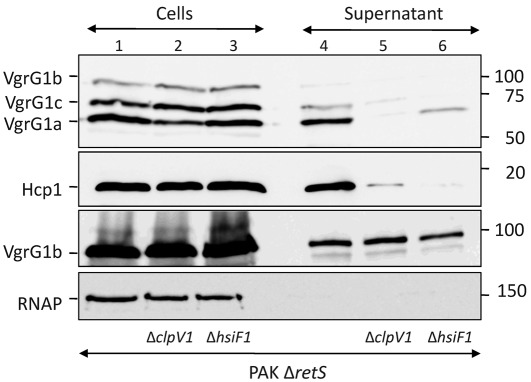
HsiF1 is essential for H1-T6SS function. PAKΔ*retS*, PAKΔ*retS*Δ*clpV1* and PAKΔ*retS*Δ*hsiF1* were grown in TSB for 5 h. Whole-cell lysates (lanes 1–3) and 10× concentrated *Pseudomonas* culture supernatant (lanes 4–6) were analysed by SDS-PAGE and Western blotting using polyclonal antibodies directed against VgrG1a (first row), Hcp1 (second row) or VgrG1b (third row). Monoclonal anti-RNAP antibody was used to control for cell lysis in the supernatant fraction (fourth row). Molecular mass markers are indicated on the right (kDa). The positions of VgrG1a, VgrG1b, VgrG1c, Hcp1 and RNAP are indicated on the left.

Generally, *T6SS* gene clusters are not expressed in laboratory conditions, but are induced in the host (Das *et al.*, 2000; Mattinen *et al.*, 2007; Parsons & Heffron, 2005). The *P. aeruginosa H1-T6SS* cluster has been extensively studied, since its expression is induced in a mutant deleted for the *retS* gene (PAKΔ*retS*), which encodes a sensor kinase (Mougous *et al.*, 2006). The H1-T6SS system has been shown to be involved in secretion of Hcp1 (PA0085), VgrG1a (PA0091), VgrG1c (PA2685) (Hachani *et al.*, 2011; Hood *et al.*, 2010; Mougous *et al.*, 2006) and the Tse1–3 proteins (Hood *et al.*, 2010). Among the 22 genes of the *H1-T6SS* cluster, only *clpV1* has been shown to be required for Hcp, VgrG and Tse secretion. To identify the role of HsiF1 in the T6SS, a deletion of the *hsiF1* gene was engineered in a PAKΔ*retS* background, resulting in PAKΔ*retS*Δ*hsiF1.* Secretion of VgrG1a, VgrG1b and VgrG1c as well as Hcp1 was analysed by SDS-PAGE and immunoblotting (Fig. 2). VgrG1a, VgrG1b and VgrG1c were detected in PAKΔ*retS* cell lysate by an antibody raised against VgrG1a (Hachani *et al.*, 2011), confirming induction of the *H1-T6SS* genes (Fig. 2, top panel, lane 1). Using this antibody, only VgrG1a and VgrG1c, but not VgrG1b, were detectable in culture supernatants (Fig. 2, top panel, lane 4), which is in agreement with our previous study (Hachani *et al.*, 2011). The deletion of *hsiF1* severely reduced secretion of VgrG1a and VgrG1c, which were poorly recovered in the culture supernatant of PAKΔ*retS*Δ*hsiF1* (Fig. 2, top panel, lane 6). Secretion of Hcp1 was also abolished in the absence of *hsiF1* (Fig. 2, second panel, lane 6), whereas Hcp1 was detectable in the cells (Fig. 2, second panel, lane 3). We analysed VgrG1b secretion using an antibody raised against VgrG1b (Hachani *et al.*, 2011). This antibody recognizes not only intracellular but also extracellular VgrG1b (Hachani *et al.*, 2011). In contrast to VgrG1a and VgrG1c, VgrG1b secretion was unaffected by deletion of *hsiF1* (Fig. 2, third panel, lane 6). This observation is in agreement with our previous report, which showed that secretion of VgrG1b is T6SS-independent, as it is still secreted in a *clpV1* deletion mutant (Fig. 2, third panel, lane 5) and a *clpV1/2/3* triple mutant (Hachani *et al.*, 2011; Hood *et al.*, 2010). In all cases, we assessed the absence of cell lysis by monitoring the localization of RNAP (Fig. 2, bottom panel). In conclusion, our data demonstrate that HsiF is an essential component for T6SS-dependent secretion in *P. aeruginosa*.

### Structure prediction for HsiF proteins

Information about HsiF structure was obtained using bioinformatic tools such as the Phyre fold prediction software (http://www.sbg.bio.ic.ac.uk/phyre). The program confirmed that HsiF proteins resemble the solved structure of the gp25-like protein encoded by *GSU0986* on the *G. sulfurreducens* genome. On this bacterial genome, two genes can be identified that encode gp25-like proteins: *GSU0986* (encoding NP_952040.1) and *GSU0430*. While *GSU0430* is part of a characteristic *T6SS* cluster (locus AE017080A), *GSU0986* is not associated with *T6SS* genes. Instead, several phage-related genes can be identified in the vicinity of *GSU0986*, such as *GSU0983*, annotated as a phage-related baseplate assembly protein, and *GSU0987*, annotated as a baseplate J-like protein found in the P2 bacteriophage. Thus, *GSU0986* encodes a gp25-like protein, which is more closely related to gp25 of the T4 phage than to gp25-like components encoded by *T6SS* gene clusters. This observation is supported by the lack of the YGL motif in GSU0986 (Fig. 1a) and its close phylogenetic relationship with gp25 of the T4 phage (Fig. 1b).

**Fig. 3.**
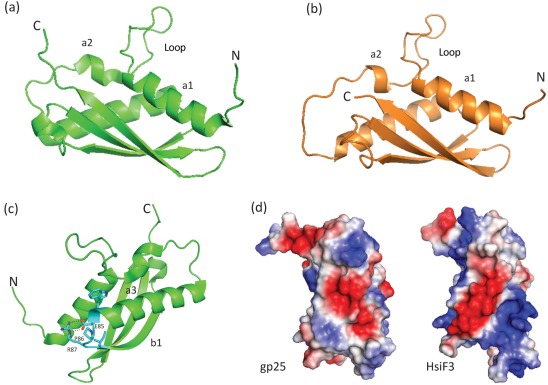
Homology modelling of HsiF homologues based on the crystal structure of GSU0986 of *G. sulfurreducens*. (a) Ribbon representation of the crystal structure of *Geobacter* GSU0986 (PDB2ia7), showing the unusual loop conformation between α1 and α2. N and C termini are labelled. (b) Ribbon representation of the HsiF3 homology model based on GSU0986 as an exemplar of the HsiF homologues. (c) Ribbon representation of GSU0986, highlighting identical residues between GSU0986 and HsiF homologues. The E85-P86-R87 forms a tight turn between α3 and β1, with E85 and R87 forming a salt bridge (dotted black line). This turn is essential for stabilizing the positions of α1, α3 and β1 and in establishing the overall gp25 fold. Other conserved residues not labelled include W84 and I88, both of which form hydrophobic interactions that also stabilize the GSU0986 fold. (d) Representations of the electrostatic surface potential for GSU0986/gp25 and HsiF3, with positive potential shown in blue and negative in red. Note the striking striated pattern of positive and negative surface potential for HsiF3 versus GSU0986/gp25. All figures were made using PyMOL (http://www.pymol.org).

The predictions for the tertiary structure of HsiF1, HsiF2 and HsiF3 were obtained from fold recognition algorithms with high statistical significance (95–100 % precision, Supplementary Fig. S2). The gp25-like protein from *Geobacter*, which we refer to as GSU0986, adopts a compact fold and belongs to the α/β fold family (Fig. 3a, c). The GSU0986 fold consists of a three-stranded anti-parallel β-sheet with one side surrounded by two long anti-parallel α-helices and a third smaller α-helix. Homology models of HsiF proteins, such as HsiF3 (Fig. 3b), are very similar to the GSU0986 structure. However, the HsiF1 structure prediction is slightly more divergent, especially within the β-domain, where two β-strands instead of three are predicted (Supplementary Fig. S2). This is likely due to amino acid sequence insertions in HsiF1 compared with HsiF2 and HsiF3. Another striking feature in the fold of the GSU0986 and HsiF proteins is an exposed loop structure that links α1 and α2 (Fig. 3a, b), which generates a triangular-shaped structure of dimensions ~42 Å×~33 Å×~22 Å.

The main sequence identities between the HsiF homologues, T4 phage gp25 and GSU0986 are residues E85, P86 and R87 (gp25-like GSU0986 numbering) (Fig. 1a). These residues form part of a short, tight turn between α3 and β1, with E85 forming a salt bridge with R87, stabilizing the position of α1 and the base of the loop (Fig. 3c). Other sequence identities relate to conserved hydrophobic core residues. In terms of surface features, an electrostatic surface representation of the homology models compared with GSU0986 shows some significant differences; for example, the external surface of the β-sheet, where HsiF3 shows a striking striated surface of negative and positive potential compared with GSU0986 (Fig. 3d). These differences could relate to different adaptations of HsiF homologues to specifically associate with their respective T6SSs.

### A conserved glutamate residue is essential for HsiF function

**Fig. 4.**
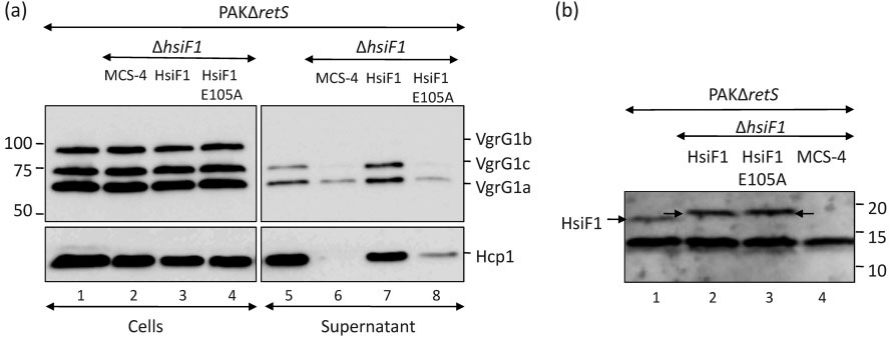
The conserved glutamate residue E105 is required for HsiF1 function. (a) PAKΔ*retS* (lanes 1 and 5), PAKΔ*retS*Δ*hsiF*1 strains containing pBBR1MCS-4 (labelled MCS-4, lanes 2 and 6), pHsiF1 (lanes 3 and 7) and pHsiF1_E105A_ (lanes 4 and 8), as indicated, were grown to late exponential phase. Whole-cell lysates (lanes 1–4) and 10× concentrated *Pseudomonas* culture supernatant (lanes 5–8) were analysed by SDS-PAGE and Western blotting using polyclonal anti-VgrG1a and anti-Hcp1 antibodies. Molecular mass markers are shown on the left (kDa). (b) HsiF1 and HsiF1_E105A_ are produced at similar levels in *P. aeruginosa*. PAKΔ*retS* (lane 1) and PAKΔ*retS*Δ*hsiF*1 containing pBBR1MCS-4 (lane 2), pHsiF1 (lane 3) or pHsiF1_E105A_ (lane 4) were grown to late exponential phase, and production of HsiF1 and HsiF1_E105A_ was analysed by Western blotting using polyclonal anti-HsiF1 antibody. Chromosomal levels of HsiF1 as well as HsiF1 and HsiF1_E105A_ produced from pHsiF1 and pHsiF1_E105A_, respectively, are indicated by arrows. HsiF1 and HsiF1_E105A_ contain a C-terminal 6×His tag, and the corresponding band therefore runs slightly more slowly than the chromosomally expressed HsiF. Molecular mass markers are indicated on the right (kDa). The band below the 15 kDa marker is a non-specific cross-reacting protein.

The conservation of a glutamate residue within the EPRL motif of the gp25 family, and its predicted role in the gp25-like GSU0986 structure (E85) to form a salt bridge with R87, supports the importance of this residue in protein function. To assess the role of the E105 residue in HsiF1, a mutation was introduced into the *hsiF1* gene, resulting in the E105A substitution. The new *hsiF1* allele, encoding HsiF1_E105A_, was cloned in pBBR1MCS-4, yielding pHsiF1_E105A_. The wild-type allele of the *hsiF1* gene was also cloned in the broad-host-range expression vector pBBR1MCS-4, yielding pHsiF1. A complementation experiment using pHsiF1 readily restored secretion of VgrG1a and VgrG1c, as well as Hcp1, in the PAKΔ*retS*Δ*hsiF1* mutant strain (Fig. 4a). In contrast, production of HsiF1_E105A_ from pHsiF1_E105A_ did not fully restore secretion of Hcp1, VgrG1a or VgrG1c (Fig. 4a). Both HsiF1 and HsiF1_E105A_ were produced at similar levels, as demonstrated by Western blot analysis using an antibody directed against HsiF1 (Fig. 4b). In conclusion, our data showed that the E105 residue is essential for HsiF1 function, as could be predicted from its central position in the HsiF fold (Fig. 3c).

### HsiF proteins do not display detectable lysozyme activity

In 1986, Szewczyk and co-workers identified gp25 of the T4 phage as a 15 kDa component responsible for lysozyme activity in cells infected with a T4 bacteriophage (Szewczyk *et al.*, 1986). Since then, proteins annotated as members of this family (gene 25-like lysozyme; pfam04965) have been assumed to possess lysozyme activity, but no further experimental evidence has supported this assumption to date. We investigated whether HsiF proteins, which are members of the gp25-like protein family, exhibit lysozyme activity *in vitro* using various approaches. All three *P. aeruginosa hsiF* genes were cloned in plasmids pET28a and pGEX4T1 (Supplementary Table S2, see Supplementary Methods), allowing the production of HsiF proteins carrying an N-terminal His or GST tag, respectively. The recombinant plasmids were introduced into *E. coli* Rosetta2 BL21(DE3). Cells were grown and lysed, and the soluble fraction was isolated by centrifugation, as described in Methods. The soluble and insoluble fractions were analysed by SDS-PAGE and Coomassie staining (Supplementary Fig. S1). In all cases, HsiF1 and HsiF2 remained mainly in the insoluble fraction, whereas HsiF3 proved to be soluble (Supplementary Fig. S1b). It is worth mentioning that the most insoluble protein, HsiF1, displayed significant structural variability in the β-domain as compared with HsiF2 and HsiF3 (Supplementary Fig. S2), which could explain its different biochemical behaviour.

**Fig. 5.**
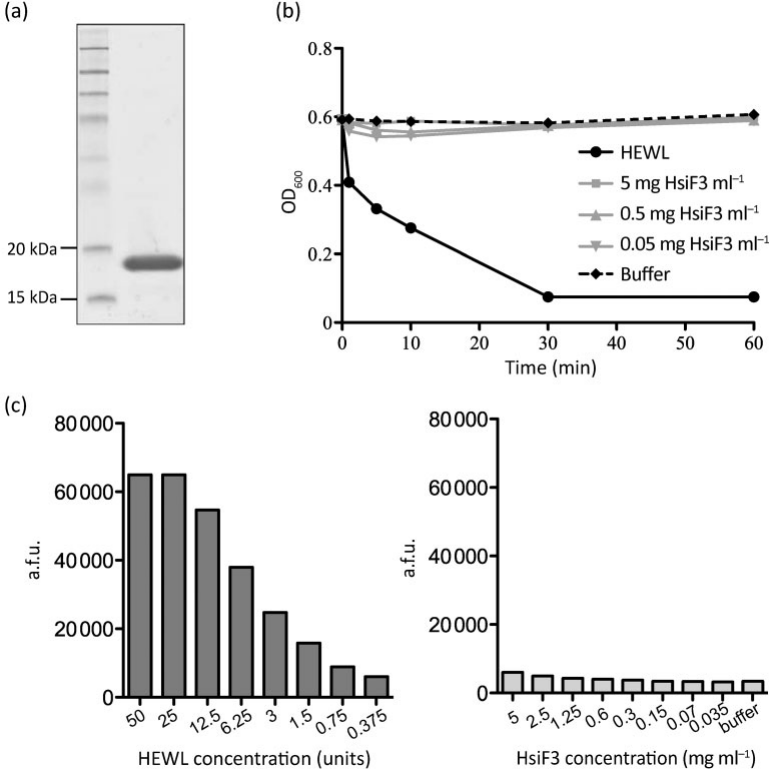
Purified HsiF3 protein does not exhibit detectable lysozyme activity. (a) Recombinant HsiF3 used for *in vitro* analysis. HsiF3 was expressed and purified as described in Methods. Purified HsiF3 was analysed by SDS-PAGE and stained by Coomassie blue. (b) Recombinant HsiF3 (5, 0.5 or 0.05 mg ml^−1^), HEWL (0.5 mg ml^−1^) or assay buffer was incubated with a suspension of *M. lysodeikticus* in 96-well plates. The decrease in turbidity was monitored at 595 nm over 60 min at 37 °C. (c) HEWL (bar chart to the left; dilution series from left to right 50 to 0.375 U per assay point) or recombinant HsiF3 (bar chart to the right; dilution series 5 to 0.035 mg ml^−1^ final enzyme concentration per assay point) was incubated with fluorescently labelled *M. lysodeikticus* for 60 min at 37 °C before fluorescence was measured as described in Methods. Emission of fluorescence is represented by arbitrary fluorescence units (a.f.u.). One unit of HEWL is defined by the manufacturer as the amount of enzyme required to produce a change in the *A*_450_ of 0.001 U min^−1^ at pH 6.24 and 25 °C, using a suspension of *M. lysodeikticus* as the substrate.

Further biochemical analyses were carried out using GST–HsiF3, which was purified with a high yield by affinity chromatography (Fig. 5a, see Methods). The GST moiety was removed using thrombin, and the potential lysozyme activity of HsiF3 was analysed *in vitro* using a suspension of freeze-dried *M. lysodeikticus* (Fig. 5b). HEWL was used as a positive control in all assays. Lysozyme activity resulting in cell lysis was monitored as the decrease in turbidity of the enzyme/substrate mixture (Fig. 5b). As expected, 0.5 mg HEWL ml^−1^ cleared the *M. lysodeikticus* suspension within 30 min (Fig. 5b). However, incubation of substrate with concentrations of recombinant HsiF3 ranging from 0.05 to 5 mg ml^−1^ did not result in a decrease of turbidity over an incubation time of 60 min, indicating that recombinant HsiF3 does not exhibit lytic activity.

Since the strength of lysozyme activity per milligram of HsiF is unknown and a weak lytic activity of HsiF3 might have been missed in the previous assay, a more sensitive assay using fluorescently labelled *M. lysodeikticus* was performed, recording fluorescence emission as a measure of activity (Fig. 5c). While an increase in fluorescence was recorded when the substrate was incubated with increasing concentrations of HEWL, no change in fluorescence was detectable after incubation of HsiF3 with labelled *M. lysodeikticus* for 60 min.

If HsiF proteins had lysozyme activity, the *P. aeruginosa* peptidoglycan would be the most likely substrate for these proteins. Accordingly, chloroform extracts of *P. aeruginosa* peptidoglycan were prepared and used as a potential substrate for both HEWL and HsiF3. In agreement with our previous findings, no hydrolysis of *P. aeruginosa* peptidoglycan was observed with HsiF3 in contrast to HEWL (Supplementary Fig. S3).

**Fig. 6.**
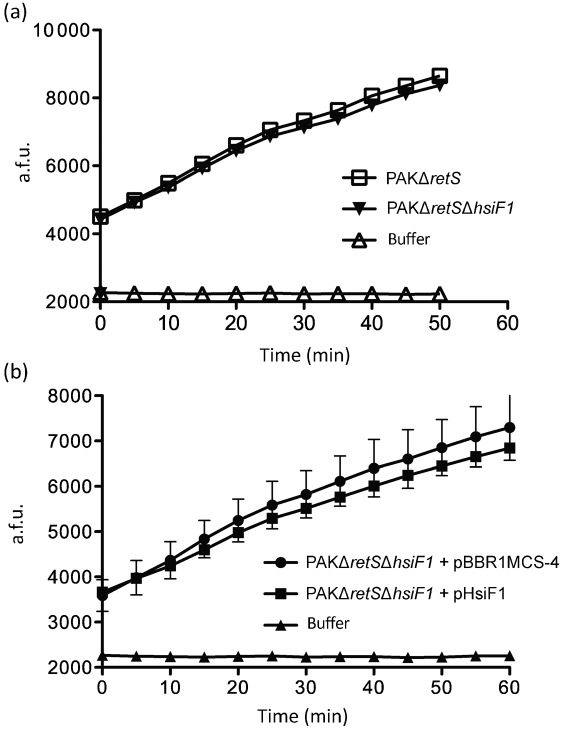
HsiF1 does not exhibit detectable lysozyme activity when expressed in *P. aeruginosa*. (a) Cell lysates of *P. aeruginosa* PAKΔ*retS* and PAKΔ*retS*Δ*hsiF1*, grown to late exponential phase as described in Methods, were incubated with fluorescently labelled *M. lysodeikticus* for 50 min at 37 °C, during which fluorescence was measured every 5 min. Assay buffer was incubated with *M. lysodeikticus* as a negative control. Emission of fluorescence is represented by arbitrary fluorescence units (a.f.u.). (b) Cell lysates of *P. aeruginosa* PAKΔ*retS*Δ*hsiF1* containing pBBR1MCS-4 or pHsiF1, grown to late exponential phase, were incubated with fluorescently labelled *M. lysodeikticus* for 60 min at 37 °C, during which fluorescence was measured every 5 min. Assay buffer was incubated with *M. lysodeikticus* as a negative control. Error bars, sd derived from triplicates of each assay point. Emission of fluorescence is represented by a.f.u.

Since it could not be excluded that HsiF3 activity was lost during the purification procedure, extracts of PAKΔ*retS* (induced production of HsiF1) and PAKΔ*retS*Δ*hsiF1* (no HsiF1 produced) were compared for lysozyme activity using fluorescently labelled *M. lysodeikticus* (Fig. 6). An increase of detectable fluorescence was observed over time with extracts from both strains compared with incubation with the assay buffer alone. However, no difference in fluorescence was observed between extracts of PAKΔ*retS* and PAKΔ*retS*Δ*hsiF1*, suggesting that the observed activity was HsiF1-independent (Fig. 6a). In order to increase the amount of HsiF1 produced in *P. aeruginosa*, we introduced the *hsiF1*-overexpressing plasmid pHsiF1 into PAKΔ*retS*Δ*hsiF1*. Lysozyme activity in extracts of this strain was compared with that of extracts of PAKΔ*retS*Δ*hsiF1* carrying the empty vector, using fluorescently labelled *M. lysodeikticus*, but no significant difference was detected (Fig. 6b). We thus concluded that it is unlikely that HsiF proteins exhibit lysozyme activity.

Finally, we also assessed the ability of HsiF proteins to hydrolyse the peptidoglycan in the periplasm of *E. coli* by engineering fusions of *hsiF1*, *hsiF2* and *hsiF3* to the *malE* gene, encoding maltose-binding protein (MBP). These constructions were made using the pMALp2x vector (periplasmic expression) and the pMALc2x vector (cytoplasmic expression) and were introduced into *E. coli* (see Supplementary Methods). In similar experiments, Russell and collaborators have shown that the *P. aeruginosa* lytic proteins Tse1 and Tse3 severely affect *E. coli* growth (Russell *et al.*, 2011). Here we showed that neither of the HsiF proteins had a lytic activity when targeted to the *E. coli* periplasm, since the growth curve of the recombinant strains was similar to the growth of the strain containing the cloning vector or strains expressing HsiF proteins in the cytosol (Supplementary Fig. S4).

### HsiF1 localizes to the cytoplasm of *P. aeruginosa*

**Fig. 7.**
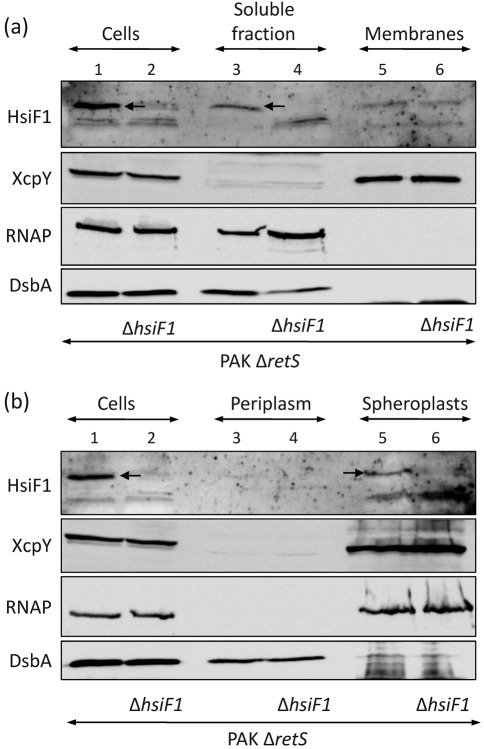
HsiF1 does not localize to the periplasm of *P. aeruginosa.* Subcellular localization of HsiF1 was analysed in PAKΔ*retS* and PAKΔ*retS*Δ*hsiF1* grown to late exponential phase. Cell fractions were analysed by SDS-PAGE and immunoblotting. Successful fractionation was verified using antibodies directed against periplasmic DsbA (bottom row), the inner-membrane protein XcpY (second row) and cytosolic RNAP (third row). HsiF1 was detected using polyclonal anti-HsiF1 antibody (first row). (a) HsiF1 localizes to the soluble fraction in *P. aeruginosa*. The soluble (lanes 3 and 4) and insoluble fractions (indicated as membranes, lanes 5 and 6) of PAKΔ*retS* and PAKΔ*retS*Δ*hsiF1* were obtained by sonication followed by ultracentrifugation as described in Methods, and were analysed by immunoblotting using the controls described above. (b) HsiF1 does not localize to the periplasm in *P. aeruginosa*. Western blot analysis of whole-cell lysate (lanes 1 and 2), periplasmic fraction (lanes 3 and 4) and spheroplasts (representing a combination of cytosol and bacterial membranes; lanes 5 and 6) was performed using the antibodies described above.

The HsiF proteins do not contain predicted transmembrane domains or signal peptides, which suggests that they are cytoplasmic proteins. The subcellular localization of HsiF1 was analysed in PAKΔ*retS*, i.e. the soluble and membrane fractions of PAKΔ*retS* and PAKΔ*retS*Δ*hsiF1* were separated after cell lysis, and protein content was analysed by SDS-PAGE and immunoblotting. The HsiF1 protein is lacking in the *hsiF1* mutant, and was recovered in the soluble fraction of PAKΔ*retS* (Fig. 7a, top panel, lane 3). Similarly, the cytoplasmic RNAP and the periplasmic DsbA (Urban *et al.*, 2001) proteins were recovered in the soluble fraction (Fig. 7a, third and fourth panels, lanes 3 and 4). In contrast, the polytopic inner-membrane protein XcpY (Bleves *et al.*, 1996) was exclusively found in the membrane fraction (Fig. 7a, second panel, lanes 5 and 6). Since HsiF1 is found in the soluble fraction, we further tested whether HsiF1 is a cytoplasmic or periplasmic protein. Spheroplasts of PAKΔ*retS* and PAKΔ*retS*Δ*hsiF1* were prepared, the periplasmic fraction was isolated, and the protein content was analysed by SDS-PAGE and immunoblotting (Fig. 7b). No cell lysis or periplasmic leakage had occurred during the fractionation procedure, since RNAP was found associated with the spheroplasts (Fig. 7b, third panel, lanes 5 and 6) and DsbA with the periplasm (Fig. 7b, bottom panel, lanes 3 and 4). We observed that HsiF1 was associated with the spheroplasts (Fig. 7b, top panel, lane 5) and that no HsiF1 protein was detectable in the periplasm (Fig. 7b, top panel, lane 3).

In order to further support the hypothesis of the cytoplasmic localization of HsiF1, an *hsiF1* : : *phoA* fusion was engineered using the pPHO7 vector (Gutierrez & Devedjian, 1989). The recombinant plasmid was introduced into the PAKΔ*retS* strain, and alkaline phosphatase activity was monitored on plates containing the alkaline phosphatase substrate 5-bromo-4-chloro-3-indolyl phosphate (BCIP). The *P. aeruginosa* colonies harbouring pHsiF1 : : phoA remained white on this plate, suggesting that HsiF1 was not able to target PhoA to the *P. aeruginosa* periplasm. Instead, the strain containing a *dsbA* : : *phoA* fusion resulted in blue colony formation (Supplementary Fig. S5). DsbA is a known periplasmic protein (Urban *et al.*, 2001). Finally, a strain encoding an *hcp1* : : *phoA* fusion also displayed blue colonies on these plates (Supplementary Fig. S5). Hcp1 has been shown to localize within most of the *P. aeruginosa* subcellular compartments, including the periplasm (Mougous *et al.*, 2007).

In Gram-negative bacteria, the peptidoglycan, which is a well-described substrate for lysozymes, is located in the periplasm. We thus concluded that HsiF1 is a cytoplasmic protein and therefore has no access to periplasmic peptidoglycan, which is in agreement with the lack of detectable lysozyme activity.

## Discussion

The discovery of a novel secretion system in Gram-negative bacteria, now referred to as the T6SS, was earlier associated with the identification of two genes encoding inner-membrane proteins with similarity to IcmF and DotU (Das & Chaudhuri, 2003). These two proteins have been described as involved in the function of another secretion system, namely the type IV secretion system (T4SS). However, the newly identified *icmF* and *dotU*-like genes belonged to gene clusters with no other similarity to *T4SS* genes. Therefore, the set of 12–15 conserved genes associated with the *icmF*/*dotU*-like genes constituted the *T6SS* clusters. Among these, one gene was identified as a *clpV*-like gene encoding a member of the AAA^+^ ATPase family. Several other genes encode proteins with no characteristic features, such as a transmembrane domain, a signal peptide or anything suggesting that they may be extracytoplasmic proteins. Finally, one gene encodes a putative outer-membrane lipoprotein, whose localization was later confirmed in enteroaggregative *E. coli* (Aschtgen *et al.*, 2008).

A significant step forward in the conceptualization of the T6SS mechanism came from structural predictions, which identified similarities between two core components of the T6SS, VgrG and Hcp, and components of the bacteriophage T4 tail structure. In brief, the T4 phage is composed of a head that contains the genetic material. The phage tail includes a tube through which DNA passes during injection. A tail sheath surrounds the tail tube and generates the contraction that promotes the DNA injection. At the tip of the tube, a hub, or tail spike, is found, which is known to contribute to the puncturing of the bacterial membrane, allowing the tail tube to penetrate into the cell. Finally, the tail spike is surrounded by a structure called the wedge, and together these form the baseplate. All the parts described above are made through the assembly of a large number of phage gene products (gp) assembled as homo- or hetero-multimeric complexes (Rossmann *et al.*, 2004). The T4 phage assembly process is an ordered mechanism, in which the formation of the baseplate is required for further assembly and polymerization of the tail tube (polymer of gp19) and the tail sheath (polymer of gp18) (Leiman *et al.*, 2010). The assembly of the baseplate follows a specific order. For example, the baseplate wedge is made of seven different proteins, namely gp11, gp10, gp7, gp8, gp6, gp53 and gp25, which interact sequentially in the order indicated here (Yap *et al.*, 2010). Furthermore, six assembled wedges will spontaneously associate to surround the hub, which includes among others gp27 and gp5, resulting in the formation of a hexagonal baseplate (Kostyuchenko *et al.*, 2003).

The T6SS component Hcp has been shown to display structural homology to the tail tube component gp19, and assembles into an equivalent structure *in vitro* (Ballister *et al.*, 2008; Leiman *et al.*, 2009). The VgrG protein has been proposed to be a combination of gp5 and gp27, which form the puncturing device of the T4 phage hub (Leiman *et al.*, 2009; Pukatzki *et al.*, 2007). The trimeric assembly of gp27–gp5 results in the formation of a so-called cell-puncturing device. It should be noted that gp5 has three domains, which are from the N to the C terminus the oligosaccharide-binding fold (OB fold), a lysozyme domain and a C-terminal β-helix domain. In VgrG proteins, the gp5-like region lacks the lysozyme domain, and the predicted OB fold is directly fused to the β-helix domain. Importantly, in case of the T4 phage, the lysozyme domain of gp5 is required for the entry process in hydrolysing the peptidoglycan contained in the periplasm and subsequently allowing penetration of the tail tube (Takeda *et al.*, 1998). This localized disruption of the peptidoglycan does not result in cell lysis and therefore the phage can inject DNA in a living cell that will support multiplication and assembly of phage progenies. Once the progenies are assembled, another phage lysozyme, encoded by gene *e* (Matthews & Remington, 1974), is responsible for global peptidoglycan hydrolysis, cell burst and release of newly synthesized virions.

A hypothesis that has resulted from these observations is that the T6SS is an inverted puncturing device, in which VgrG proteins could be used to perforate the bacterial cell envelope from the inside to the outside, allowing elongation of the Hcp tube and the release of genuine substrates into the extracellular medium. This hypothesis is attractive, since no integral outer-membrane components have been identified in the T6SS and using a puncturing mechanism will thus bypass the requirement for an outer-membrane channel. If the puncturing of the bacterial cell envelope involves VgrG, the lack of the lysozyme-like domain in these proteins, as compared with the T4 phage gp5 protein, prompted us to hypothesize that another component of the T6SS system could play this role. A study from 1986 reported that the T4 phage baseplate component gp25 is a 15 kDa lysozyme (Nakagawa *et al.*, 1985; Szewczyk *et al.*, 1986). In those studies it was found that lysozyme activity could be purified from bacterial cells infected with phage particles. When comparing the occurrence of this activity in various genetic backgrounds, a 15 kDa lyzosyme-dependent activity was still found in extract resulting from infection with phages mutated in gene *e* or gene *5*, but this activity was lost in a mutant in gene *25*.

In the present study, we confirmed that one of the conserved T6SS components, exemplified here by HsiF from *P. aeruginosa*, belongs to the gp25 family. However, we showed in a variety of assays that HsiF does not exhibit lysozyme activity. In order to further address this issue, we hypothesized that if HsiF had any lysozyme activity it should be targeted to the peptidoglycan-containing periplasm. Interestingly, we observed that HsiF1 is located in the cytoplasmic fraction, out of reach of an interaction with the peptidoglycan. From these data, we suggest that HsiF-like proteins, and likely the gp25 family, do not possess lysozyme activity. Although HsiF does not seem to interact with the peptidoglycan, it has been shown that other T6SS components play a role in assembly of the secretion system into the bacterial cell wall, in particular in anchoring the secretion system to the peptidoglycan layer (Aschtgen *et al.*, 2010a, b).

Importantly, it has been shown that gp25 is a structural component of the phage tail, and that the formation of the baseplate structure requires gp25 (Yap *et al.*, 2010), which is likely to be independent of any putative lysozyme activity. In the predicted structure of HsiF proteins, we observed that a conserved glutamate residue is central to establish a salt bridge stabilizing the HsiF fold. By substituting the glutamate residue with alanine, we obtained a recombinant protein that was not able to restore secretion in an *hsiF* mutant. This observation further supports the contention that HsiF is an essential structural component of the T6SS, likely similar to gp25 in phage baseplate formation.

The structural similarity of VgrG, Hcp and HsiF to gp27–gp5, gp19 and gp25, respectively, suggests that additional resemblance may exist between the T6SS and the T4 phage structure. It has been proposed, for example, that VipA and VipB from the *V. cholerae* T6SS, which are the *P. aeruginosa* HsiB and HsiC homologues (Filloux *et al.*, 2008), also called TssB and TssC (Hood *et al.*, 2010), respectively, may have a function related to gp18 that constitutes the bacteriophage tail sheath (Bönemann *et al.*, 2010). This is based more on functional rather than on structural aspects. Indeed, VipA/B have been shown to be able to form microtubules, whose assembly/disassembly is dependent on ClpV ATPase activity. This process may be compared with the contractile property of the tail sheath made by the helical arrangement of about 150 subunits of gp18 (Kostyuchenko *et al.*, 2003).

Our future work will concentrate on elucidating the existing protein–protein interaction between HsiF and other components of the T6SS, and try to correlate these interactions with the known interaction between gp25 and other components of the baseplate such as gp53 (Yap *et al.*, 2010). No other obvious homologies could be detected between T6SS components and gp proteins, and we will attempt to solve the 3D structure of the Hsi proteins to get further insights into their role in type VI secretion and T6SS assembly.

## Supplementary Material

Supplementary material

## References

[r1] Aschtgen M. S. Bernard C. S. De Bentzmann S. Lloubès R. Cascales E. **(**2008**).** SciN is an outer membrane lipoprotein required for type VI secretion in enteroaggregative *Escherichia coli*. J Bacteriol 190, 7523–7531. 10.1128/JB.00945-08 18805985 PMC2576670

[r2] Aschtgen M. S. Gavioli M. Dessen A. Lloubès R. Cascales E. **(**2010a**).** The SciZ protein anchors the enteroaggregative *Escherichia coli* type VI secretion system to the cell wall. Mol Microbiol 75, 886–899. 10.1111/j.1365-2958.2009.07028.x 20487285

[r3] Aschtgen M. S. Thomas M. S. Cascales E. **(**2010b**).** Anchoring the type VI secretion system to the peptidoglycan: TssL, TagL, TagP... what else? Virulence 1, 535–540. 10.4161/viru.1.6.13732 21178498

[r4] Ballister E. R. Lai A. H. Zuckermann R. N. Cheng Y. Mougous J. D. **(**2008**).** In vitro self-assembly of tailorable nanotubes from a simple protein building block. Proc Natl Acad Sci U S A 105, 3733–3738. 10.1073/pnas.0712247105 18310321 PMC2268831

[r5] Bingle L. E. Bailey C. M. Pallen M. J. **(**2008**).** Type VI secretion: a beginner’s guide. Curr Opin Microbiol 11, 3–8. 10.1016/j.mib.2008.01.006 18289922

[r6] Bladergroen M. R. Badelt K. Spaink H. P. **(**2003**).** Infection-blocking genes of a symbiotic *Rhizobium leguminosarum* strain that are involved in temperature-dependent protein secretion. Mol Plant Microbe Interact 16, 53–64. 10.1094/MPMI.2003.16.1.53 12580282

[r7] Bleves S. Lazdunski A. Filloux A. **(**1996**).** Membrane topology of three Xcp proteins involved in exoprotein transport by *Pseudomonas aeruginosa*. J Bacteriol 178, 4297–4300. 8763961 10.1128/jb.178.14.4297-4300.1996 PMC178190

[r8] Bleves S. Viarre V. Salacha R. Michel G. P. Filloux A. Voulhoux R. **(**2010**).** Protein secretion systems in *Pseudomonas aeruginosa*: a wealth of pathogenic weapons. Int J Med Microbiol 300, 534–543. 10.1016/j.ijmm.2010.08.005 20947426

[r9] Bönemann G. Pietrosiuk A. Diemand A. Zentgraf H. Mogk A. **(**2009**).** Remodelling of VipA/VipB tubules by ClpV-mediated threading is crucial for type VI protein secretion. EMBO J 28, 315–325. 10.1038/emboj.2008.269 19131969 PMC2646146

[r10] Bönemann G. Pietrosiuk A. Mogk A. **(**2010**).** Tubules and donuts: a type VI secretion story. Mol Microbiol 76, 815–821. 10.1111/j.1365-2958.2010.07171.x 20444095

[r11] Cascales E. **(**2008**).** The type VI secretion toolkit. EMBO Rep 9, 735–741. 10.1038/embor.2008.131 18617888 PMC2515208

[r12] Das S. Chaudhuri K. **(**2003**).** Identification of a unique IAHP (IcmF associated homologous proteins) cluster in *Vibrio cholerae* and other proteobacteria through in silico analysis. In Silico Biol 3, 287–300. 12954091

[r13] Das S. Chakrabortty A. Banerjee R. Roychoudhury S. Chaudhuri K. **(**2000**).** Comparison of global transcription responses allows identification of *Vibrio cholerae* genes differentially expressed following infection. FEMS Microbiol Lett 190, 87–91. 10.1111/j.1574-6968.2000.tb09267.x 10981695

[r14] Filloux A. **(**2009**).** The type VI secretion system: a tubular story. EMBO J 28, 309–310. 10.1038/emboj.2008.301 19225443 PMC2646157

[r15] Filloux A. Hachani A. Bleves S. **(**2008**).** The bacterial type VI secretion machine: yet another player for protein transport across membranes. Microbiology 154, 1570–1583. 10.1099/mic.0.2008/016840-0 18524912

[r16] Goodman A. L. Kulasekara B. Rietsch A. Boyd D. Smith R. S. Lory S. **(**2004**).** A signaling network reciprocally regulates genes associated with acute infection and chronic persistence in *Pseudomonas aeruginosa*. Dev Cell 7, 745–754. 10.1016/j.devcel.2004.08.020 15525535

[r17] Gutierrez C. Devedjian J. C. **(**1989**).** A plasmid facilitating in vitro construction of *phoA* gene fusions in *Escherichia coli*. Nucleic Acids Res 17, 3999. 10.1093/nar/17.10.3999 2660110 PMC317897

[r18] Hachani A. Lossi N. S. Hamilton A. Jones C. Bleves S. Albesa-Jové D. Filloux A. **(**2011**).** Type VI secretion system in *Pseudomonas aeruginosa*: secretion and multimerization of VgrG proteins. J Biol Chem 286, 12317–12327. 10.1074/jbc.M110.193045 21325275 PMC3069435

[r19] Hauser A. R. **(**2009**).** The type III secretion system of *Pseudomonas aeruginosa*: infection by injection. Nat Rev Microbiol 7, 654–665. 10.1038/nrmicro2199 19680249 PMC2766515

[r20] Hauser A. R. Cobb E. Bodi M. Mariscal D. Vallés J. Engel J. N. Rello J. **(**2002**).** Type III protein secretion is associated with poor clinical outcomes in patients with ventilator-associated pneumonia caused by *Pseudomonas aeruginosa*. Crit Care Med 30, 521–528. 10.1097/00003246-200203000-00005 11990909

[r21] Hood R. D. Singh P. Hsu F. Güvener T. Carl M. A. Trinidad R. R. Silverman J. M. Ohlson B. B. Hicks K. G. **& other authors (**2010**).** A type VI secretion system of *Pseudomonas aeruginosa* targets a toxin to bacteria. Cell Host Microbe 7, 25–37. 10.1016/j.chom.2009.12.007 20114026 PMC2831478

[r22] Kaniga K. Delor I. Cornelis G. R. **(**1991**).** A wide-host-range suicide vector for improving reverse genetics in Gram-negative bacteria: inactivation of the *blaA* gene of *Yersinia enterocolitica*. Gene 109, 137–141. 10.1016/0378-1119(91)90599-7 1756974

[r23] Kelley L. A. Sternberg M. J. **(**2009**).** Protein structure prediction on the Web: a case study using the Phyre server. Nat Protoc 4, 363–371. 10.1038/nprot.2009.2 19247286

[r24] Kostyuchenko V. A. Leiman P. G. Chipman P. R. Kanamaru S. van Raaij M. J. Arisaka F. Mesyanzhinov V. V. Rossmann M. G. **(**2003**).** Three-dimensional structure of bacteriophage T4 baseplate. Nat Struct Biol 10, 688–693. 10.1038/nsb970 12923574

[r25] Laemmli U. K. **(**1970**).** Cleavage of structural proteins during the assembly of the head of bacteriophage T4. Nature 227, 680–685. 10.1038/227680a0 5432063

[r26] Leiman P. G. Basler M. Ramagopal U. A. Bonanno J. B. Sauder J. M. Pukatzki S. Burley S. K. Almo S. C. Mekalanos J. J. **(**2009**).** Type VI secretion apparatus and phage tail-associated protein complexes share a common evolutionary origin. Proc Natl Acad Sci U S A 106, 4154–4159. 10.1073/pnas.0813360106 19251641 PMC2657435

[r27] Leiman P. G. Arisaka F. van Raaij M. J. Kostyuchenko V. A. Aksyuk A. A. Kanamaru S. Rossmann M. G. **(**2010**).** Morphogenesis of the T4 tail and tail fibers. Virol J 7, 355. 10.1186/1743-422X-7-355 21129200 PMC3004832

[r28] Lesic B. Starkey M. He J. Hazan R. Rahme L. G. **(**2009**).** Quorum sensing differentially regulates *Pseudomonas aeruginosa* type VI secretion locus I and homologous loci II and III, which are required for pathogenesis. Microbiology 155, 2845–2855. 10.1099/mic.0.029082-0 19497948 PMC2888175

[r29] Lory S. Jin S. Boyd J. M. Rakeman J. L. Bergman P. **(**1996**).** Differential gene expression by *Pseudomonas aeruginosa* during interaction with respiratory mucus. Am J Respir Crit Care Med 154, S183–S186. 8876539 10.1164/ajrccm/154.4_Pt_2.S183

[r30] Ma A. T. McAuley S. Pukatzki S. Mekalanos J. J. **(**2009**).** Translocation of a *Vibrio cholerae* type VI secretion effector requires bacterial endocytosis by host cells. Cell Host Microbe 5, 234–243. 10.1016/j.chom.2009.02.005 19286133 PMC3142922

[r31] Matthews B. W. Remington S. J. **(**1974**).** The three dimensional structure of the lysozyme from bacteriophage T4. Proc Natl Acad Sci U S A 71, 4178–4182. 10.1073/pnas.71.10.4178 4530293 PMC434353

[r32] Mattinen L. Nissinen R. Riipi T. Kalkkinen N. Pirhonen M. **(**2007**).** Host-extract induced changes in the secretome of the plant pathogenic bacterium *Pectobacterium atrosepticum*. Proteomics 7, 3527–3537. 10.1002/pmic.200600759 17726675

[r33] Merrell D. S. Falkow S. **(**2004**).** Frontal and stealth attack strategies in microbial pathogenesis. Nature 430, 250–256. 10.1038/nature02760 15241423

[r34] Michel G. Bleves S. Ball G. Lazdunski A. Filloux A. **(**1998**).** Mutual stabilization of the XcpZ and XcpY components of the secretory apparatus in *Pseudomonas aeruginosa*. Microbiology 144, 3379–3386. 10.1099/00221287-144-12-3379 9884230

[r35] Mougous J. D. Cuff M. E. Raunser S. Shen A. Zhou M. Gifford C. A. Goodman A. L. Joachimiak G. Ordoñez C. L. **& other authors (**2006**).** A virulence locus of *Pseudomonas aeruginosa* encodes a protein secretion apparatus. Science 312, 1526–1530. 10.1126/science.1128393 16763151 PMC2800167

[r36] Mougous J. D. Gifford C. A. Ramsdell T. L. Mekalanos J. J. **(**2007**).** Threonine phosphorylation post-translationally regulates protein secretion in *Pseudomonas aeruginosa*. Nat Cell Biol 9, 797–803. 10.1038/ncb1605 17558395

[r37] Nakagawa H. Arisaka F. Ishii S. **(**1985**).** Isolation and characterization of the bacteriophage T4 tail-associated lysozyme. J Virol 54, 460–466. 3157805 10.1128/jvi.54.2.460-466.1985 PMC254817

[r38] Nakimbugwe D. Masschalck B. Deckers D. Callewaert L. Aertsen A. Michiels C. W. **(**2006**).** Cell wall substrate specificity of six different lysozymes and lysozyme inhibitory activity of bacterial extracts. FEMS Microbiol Lett 259, 41–46. 10.1111/j.1574-6968.2006.00240.x 16684100

[r39] Parsons D. A. Heffron F. **(**2005**).** *sciS*, an *icmF* homolog in *Salmonella enterica* serovar Typhimurium, limits intracellular replication and decreases virulence. Infect Immun 73, 4338–4345. 10.1128/IAI.73.7.4338-4345.2005 15972528 PMC1168621

[r40] Potvin E. Lehoux D. E. Kukavica-Ibrulj I. Richard K. L. Sanschagrin F. Lau G. W. Levesque R. C. **(**2003**).** In vivo functional genomics of *Pseudomonas aeruginosa* for high-throughput screening of new virulence factors and antibacterial targets. Environ Microbiol 5, 1294–1308. 10.1046/j.1462-2920.2003.00542.x 14641575

[r41] Pukatzki S. Ma A. T. Revel A. T. Sturtevant D. Mekalanos J. J. **(**2007**).** Type VI secretion system translocates a phage tail spike-like protein into target cells where it cross-links actin. Proc Natl Acad Sci U S A 104, 15508–15513. 10.1073/pnas.0706532104 17873062 PMC2000545

[r42] Pukatzki S. McAuley S. B. Miyata S. T. **(**2009**).** The type VI secretion system: translocation of effectors and effector-domains. Curr Opin Microbiol 12, 11–17. 10.1016/j.mib.2008.11.010 19162533

[r43] Records A. R. Gross D. C. **(**2010**).** Sensor kinases RetS and LadS regulate *Pseudomonas syringae* type VI secretion and virulence factors. J Bacteriol 192, 3584–3596. 10.1128/JB.00114-10 20472799 PMC2897360

[r44] Rossmann M. G. Mesyanzhinov V. V. Arisaka F. Leiman P. G. **(**2004**).** The bacteriophage T4 DNA injection machine. Curr Opin Struct Biol 14, 171–180. 10.1016/j.sbi.2004.02.001 15093831

[r45] Roy-Burman A. Savel R. H. Racine S. Swanson B. L. Revadigar N. S. Fujimoto J. Sawa T. Frank D. W. Wiener-Kronish J. P. **(**2001**).** Type III protein secretion is associated with death in lower respiratory and systemic *Pseudomonas aeruginosa* infections. J Infect Dis 183, 1767–1774. 10.1086/320737 11372029

[r46] Russell A. B. Hood R. D. Bui N. K. LeRoux M. Vollmer W. Mougous J. D. **(**2011**).** Type VI secretion delivers bacteriolytic effectors to target cells. Nature 475, 343–347. 10.1038/nature10244 21776080 PMC3146020

[r47] Schwarz S. Hood R. D. Mougous J. D. **(**2010**).** What is type VI secretion doing in all those bugs? Trends Microbiol 18, 531–537. 10.1016/j.tim.2010.09.001 20961764 PMC2991376

[r48] Suarez G. Sierra J. C. Erova T. E. Sha J. Horneman A. J. Chopra A. K. **(**2010**).** A type VI secretion system effector protein, VgrG1, from *Aeromonas hydrophila* that induces host cell toxicity by ADP ribosylation of actin. J Bacteriol 192, 155–168. 10.1128/JB.01260-09 19880608 PMC2798274

[r49] Szewczyk B. Bienkowska-Szewczyk K. Kozloff L. M. **(**1986**).** Identification of T4 gene 25 product, a component of the tail baseplate, as a 15K lysozyme. Mol Gen Genet 202, 363–367. 10.1007/BF00333263 3520236

[r50] Takeda S. Hoshida K. Arisaka F. **(**1998**).** Mapping of functional sites on the primary structure of the tail lysozyme of bacteriophage T4 by mutational analysis. Biochim Biophys Acta 1384, 243–252. 10.1016/S0167-4838(98)00016-8 9659385

[r51] Urban A. Leipelt M. Eggert T. Jaeger K. E. **(**2001**).** DsbA and DsbC affect extracellular enzyme formation in *Pseudomonas aeruginosa*. J Bacteriol 183, 587–596. 10.1128/JB.183.2.587-596.2001 11133952 PMC94914

[r52] Vasseur P. Vallet-Gely I. Soscia C. Genin S. Filloux A. **(**2005**).** The *pel* genes of the *Pseudomonas aeruginosa* PAK strain are involved at early and late stages of biofilm formation. Microbiology 151, 985–997. 10.1099/mic.0.27410-0 15758243

[r53] Ventre I. Goodman A. L. Vallet-Gely I. Vasseur P. Soscia C. Molin S. Bleves S. Lazdunski A. Lory S. Filloux A. **(**2006**).** Multiple sensors control reciprocal expression of *Pseudomonas aeruginosa* regulatory RNA and virulence genes. Proc Natl Acad Sci U S A 103, 171–176. 10.1073/pnas.0507407103 16373506 PMC1324988

[r54] Yap M. L. Mio K. Leiman P. G. Kanamaru S. Arisaka F. **(**2010**).** The baseplate wedges of bacteriophage T4 spontaneously assemble into hubless baseplate-like structure in vitro. J Mol Biol 395, 349–360. 10.1016/j.jmb.2009.10.071 19896486

[r55] Zheng J. Leung K. Y. **(**2007**).** Dissection of a type VI secretion system in *Edwardsiella tarda*. Mol Microbiol 66, 1192–1206. 10.1111/j.1365-2958.2007.05993.x 17986187

