# Supplementary material for: Structure–function analysis of HsiF, a gp25-like component of the type VI secretion system, in Pseudomonas aeruginosa
Source: Microbiology (Reading). 2011 Dec;157(Pt 12):3292–305. doi: 10.1099/mic.0.051987-0 (PMC3352280; doi:10.1099/mic.0.051987-0)
Supplement: Supplementary material [file supp_157_12_3292__index.html]

Supplementary data 

# Structure-function analysis of HsiF, a gp25-like component of the type VI secretion system in Pseudomonas aeruginosa

## Lossi *et al.* supplementary data

**Files in this Data Supplement:**

- Supplementary methods
- Supplementary Figs S1 - S5 legends
- Supplementary Figs S1 - S5
- Supplementary Tables S1 - S3
