# Supplementary material for: Structure–function analysis of HsiF, a gp25-like component of the type VI secretion system, in Pseudomonas aeruginosa
Source: Microbiology (Reading). 2011 Dec;157(Pt 12):3292–305. doi: 10.1099/mic.0.051987-0 (PMC3352280; doi:10.1099/mic.0.051987-0)

Structure–function analysis of HsiF, a gp25-like component of the type VI secretion system, in *Pseudomonas aeruginosa*

By: Nadine S. Lossi, Rana Dajani, Paul Freemont and Alain Filloux

SUPPLEMENTARY FIGURES

Supplementary Fig. S1

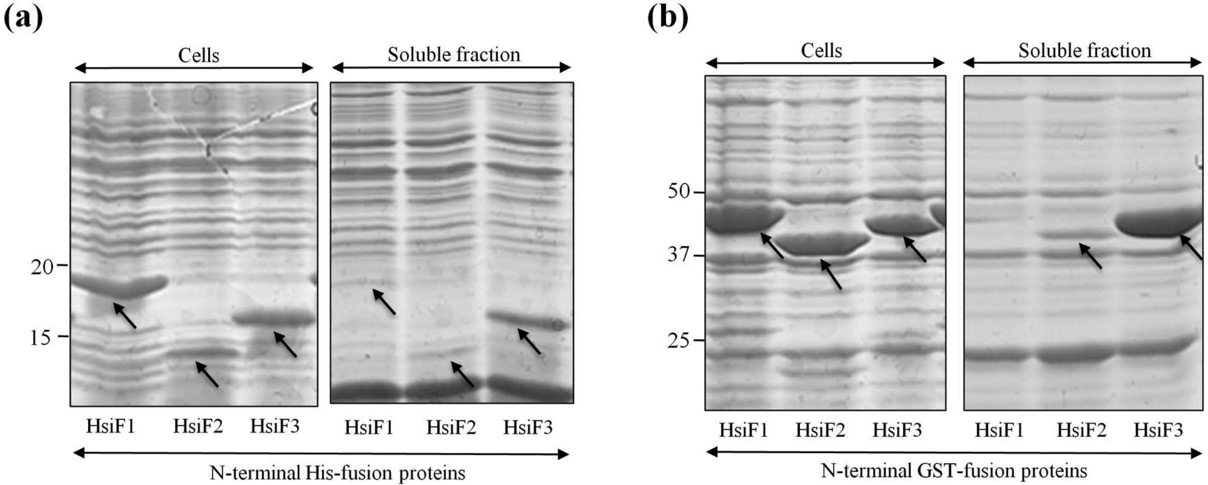

Supplementary Fig. S2

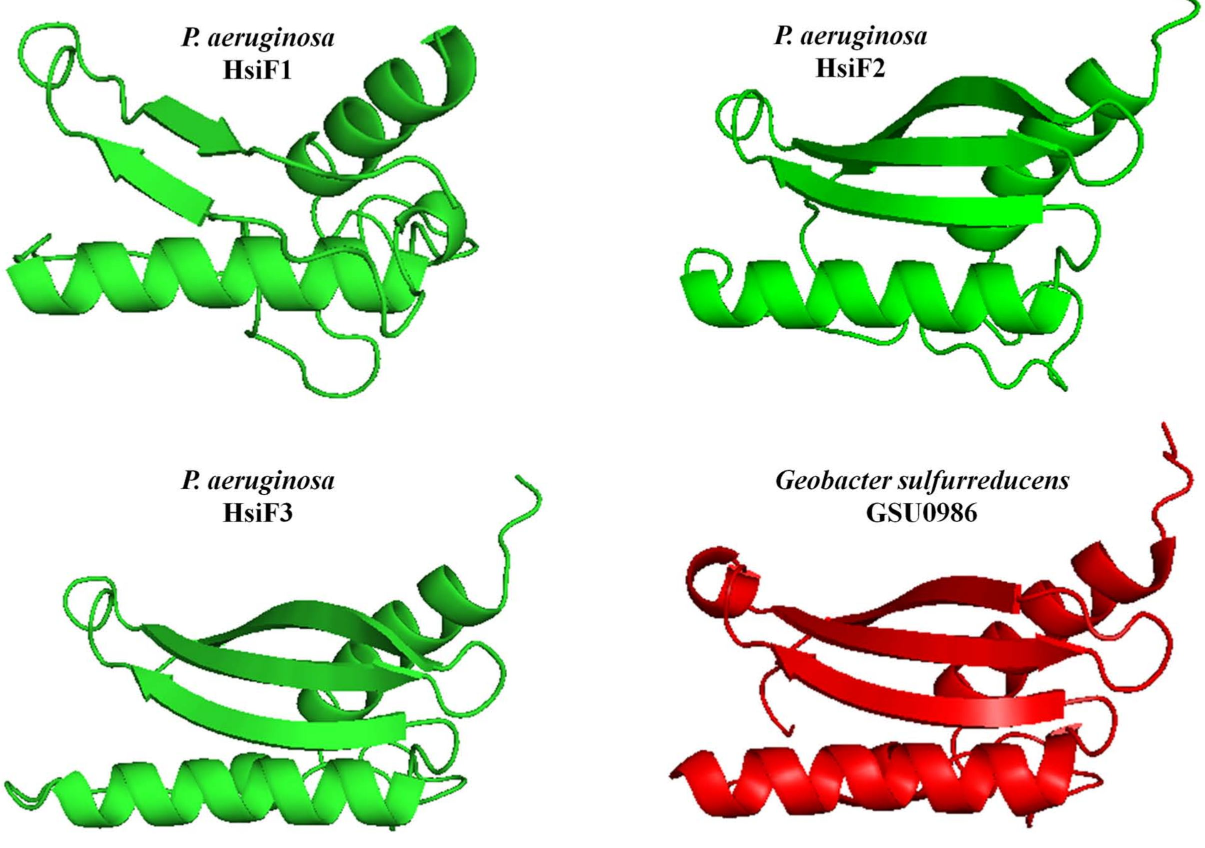

Supplementary Fig. S3

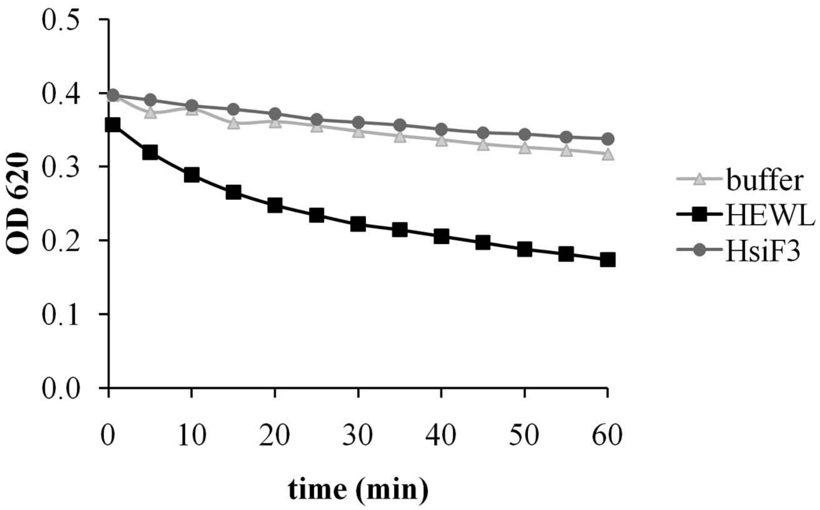

Supplementary Fig. S4

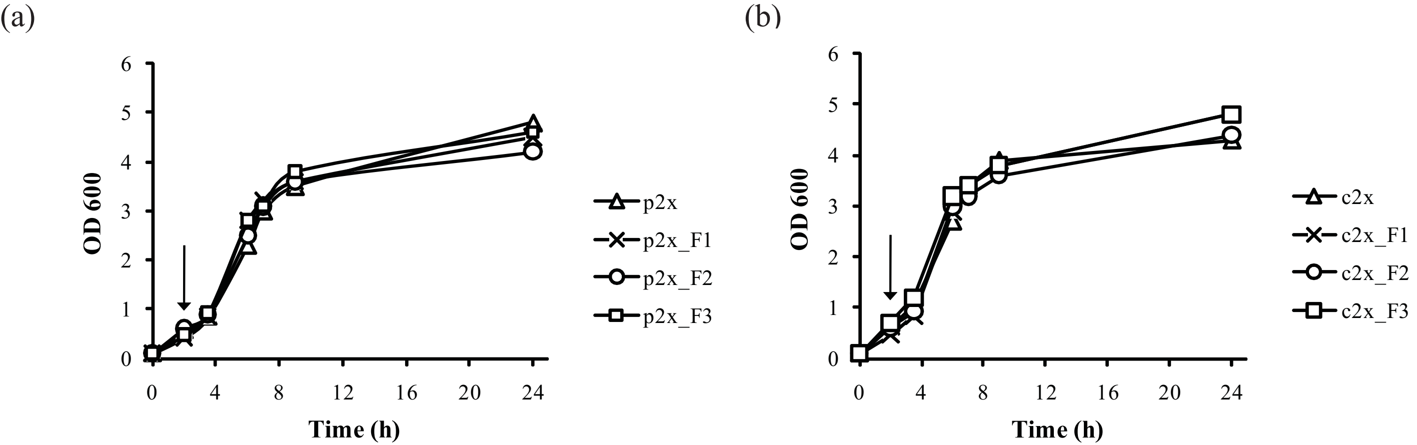

Supplementary Fig. S5

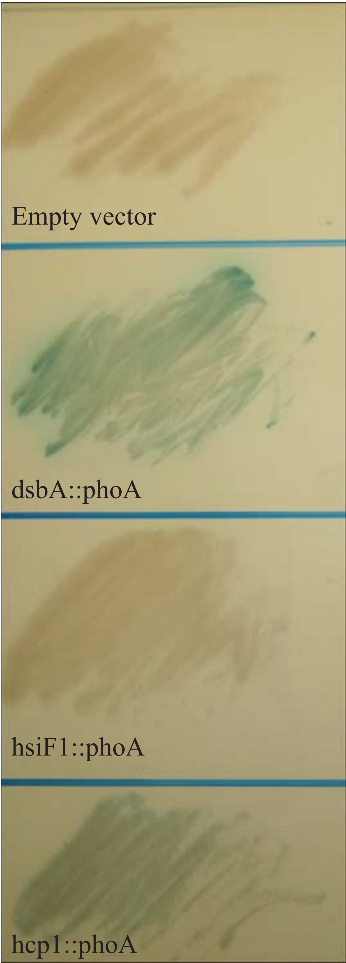

Supplement: Supplementary material [file supp_157.12.3292_mic051987_suppl_figs_S1-S5.pdf]
