# Supplementary material for: Structure–function analysis of HsiF, a gp25-like component of the type VI secretion system, in Pseudomonas aeruginosa
Source: Microbiology (Reading). 2011 Dec;157(Pt 12):3292–305. doi: 10.1099/mic.0.051987-0 (PMC3352280; doi:10.1099/mic.0.051987-0)
Supplement: Supplementary material [file supp_157.12.3292_mic051987_suppl_figs_S1-S5_legends.pdf]

Structure–function analysis of HsiF, a gp25-like component of the type VI secretion system, in *Pseudomonas aeruginosa*

By: Nadine S. Lossi, Rana Dajani, Paul Freemont and Alain Filloux

SUPPLEMENTARY FIGURE LEGENDS

Supplementary Fig. S1. Purification and solubility of HsiF proteins. Recombinant HsiF proteins were produced from pGEX-F1, pGEX-F2, pGEX-F3 pET-F1, pET-F2 and pET-F3 (Supplementary Table S1) in Rosetta2 cells grown for 16 h at 20 °C. Cells were lysed using a French press and the soluble fraction was isolated by centrifugation as described in Methods. Whole-cell lysates (Cells) and soluble fraction were analysed by SDS-PAGE and Coomassie staining. Molecular mass markers are indicated on the left (kDa). The positions of HsiF proteins are indicated by arrows. (a) Whole-cell lysate (Cells) and soluble fraction of strains producing (from left to right) GST–HsiF1 (43 kDa), GST–HsiF2 (39 kDa) or GST–HsiF3 (40 kDa); (b) whole-cell lysate (Cells) and soluble fraction of strains producing His–HsiF1 (19 kDa), His–HsiF2 (14 kDa) or His–HsiF3 (15 kDa).

Supplementary Fig. S2. Tertiary structure prediction of HsiF proteins. Tertiary structures of HsiF1, HsiF2 and HsiF3 were predicted using Phyre software based on the solved tertiary structure of the gp25-like protein of *Geobacter sulfurreducens* GSU0986. All images were made using PyMOL (http://www.pymol.org).

Supplementary Fig. S3. No detectable lysis of *P. aeruginosa* by HsiF3. The outer membrane of *P. aeruginosa* PAO1 cells was permeabilized using chloroform-saturated Tris buffer, as described in supplementary Methods. Permeabilized PAO1 cells were incubated at 37 °C with hen egg white lysozyme (HEWL), HsiF3 or assay buffer, and turbidity was monitored at 620 nm for 60 min as a measure of cell lysis.

Supplementary Fig. S4. Expression of HsiF proteins in the periplasm of *Escherichia coli* does not result in any growth defect or cell lysis. (a) The expression plasmids pMALp2x-F1, pMALp2x-F2 and pMALp2x-F3 expressing periplasmic maltose-binding protein (MBP) fusions of HsiF1, HsiF2 and HsiF3, respectively, and (b) pMALc2x-F1, pMALc2x-F2 and pMALc2x-F3 expressing cytoplasmic MBP fusions of HsiF1, HsiF2 and HsiF3, respectively, were transformed into *E. coli* XL1-blue cells. The growth rate of *E. coli* harbouring the empty vectors pMALp2x and pMALc2x served as a positive control. Protein expression was induced using 0.5 mM IPTG at the time point indicated by an arrow. Protein expression was carried out at 25 °C and OD_{600} was monitored over 20 h following protein induction.

Supplementary Fig. S5. HsiF1 does not localize to the periplasm of *P. aeruginosa*. The plasmids pDsbA::phoA, pHsiF1::phoA and pHcp1::phoA expressing fusions of DsbA, HsiF1 and Hcp1, respectively, to PhoA lacking its signal peptide were transferred into *P. aeruginosa* PAKΔ*retS* by conjugation, and conjugants were plated onto Luria–Bertani (LB) agar supplemented with 40 µg ml^{-1} of the alkaline phosphatase substrate BCIP (5-bromo-4-chloro-3-indolyl phosphate). Blue colony formation is due to PhoA activity in the periplasm and indicates periplasmic localization of the fusion protein. Localization of DsbA and Hcp1 was used as a positive control, and conjugants harbouring the empty vector pBBR1MCS-5 acted as a negative control.
