# Supplementary material for: Structure–function analysis of HsiF, a gp25-like component of the type VI secretion system, in Pseudomonas aeruginosa
Source: Microbiology (Reading). 2011 Dec;157(Pt 12):3292–305. doi: 10.1099/mic.0.051987-0 (PMC3352280; doi:10.1099/mic.0.051987-0)
Supplement: Supplementary material [file supp_157.12.3292_mic051987_suppl_methods.pdf]

Structure–function analysis of HsiF, a gp25-like component of the type VI secretion system, in *Pseudomonas aeruginosa*

By: Nadine S. Lossi, Rana Dajani, Paul Freemont and Alain Filloux

SUPPLEMENTARY METHODS

Plasmids. Plasmids used in this study are summarized in Supplementary Table S2. The plasmids pET-F2, pET-F3, pGEX-F1 and pGEX-F2 were used for protein production and purification, and constructed as follows. Using genomic *P. aeruginosa* PAO1 DNA as a template, *hsiF1*, *hsiF2* and *hsiF3* were amplified by PCR using oligonucleotide pairs OAL93/94 (pGEX-F1), OAL95/97 (pET-F2), OAl96/97 (pGEX-F2) and OAL97/99 (pET-F3) (Supplementary Table S2). Appropriate restriction sites were included in the respective ends of the PCR product for cloning purposes. PCR products were cloned into pCR2.1 (Invitrogen) and cloning was confirmed by sequencing (GATC Biotech) before subcloning into pGEX4T1 (GE Healthcare) or pET28a (Stratagene). The resulting recombinant plasmids encoded N-terminal glutathione *S*-transferase or 6×histidine fusion proteins, respectively. The plasmids pMALp2x-F1, pMALp2x-F2 pMALp2x-F3, pMALc2x-F1, pMALc2x-F2 and pMALc2x-F3 were constructed by extraction of *hsiF1*, *hsiF2* and *hsiF3* from pGEX-F1, pGEX-F2 and pGEX-F3, respectively, by restriction digestion using *Bam*HI and *Xho*I, and were ligated into pMALp2x and pMALc2x, respectively, using their *Bam*HI and *Sal*I restriction sites. The fusion constructs pDsbA::phoA, pHsiF1::phoA and pHsiF1::phoA were constructed as follows. The genes encoding DsbA (PA5489), HsiF1 (PA0087) and Hcp1 (PA0085) were amplified from genomic DNA of PAO1 *P. aeruginosa* including their native ribosome-binding site, but excluding their stop codon, using primer pairs OAL 878/888, OAL 746/890 and OAL 748/891, respectively, adding restriction sites for *Hin*dIII and *Sma*I, and were ligated into the cloning vector pCR2.1 (Invitrogen). Plasmid pPHO7 was used to extract *phoA* using restriction sites for *Sma*I and *Xho*I. The extracted *phoA* gene was then fused to *dsbA*, *hsiF1* and *hcp1*, respectively, in pCR2.1 using *Sma*I and *Xho*I. Finally, the fusions of the gene of interest to *phoA* were extracted from pCR2.1 using *Hin*dIII and *Xho*I and ligated into pBBR1MCS-5 for expression in *P. aeruginosa*.

Protein expression. The plasmids pET-F1, pET-F2, pET-F3, pGEX-F1, pGEX-F2 and pGEX-F3 were transformed into *Escherichia coli* Rosetta2 BL21(DE3) for production and purification of fusion proteins. Cells were grown at 37 °C to OD_{600} 0.6, and expression was subsequently induced using 1 mM IPTG (Sigma) for 16 h at 20 °C. Bacterial cultures were centrifuged at 4000 ***g*** for 15 min at 4 °C, cell pellets were collected in lysis buffer [50 mM HEPES, 200 mM NaCl, 5–10 mM DTT, 2 mM EDTA, supplemented with Complete Protease Inhibitor Cocktail (Roche)], and cells were lysed using a French press. The cell

extract was clarified at 40000 ***g*** for 45 min at 4 °C. To assess protein solubility the resulting pellet (insoluble fraction) and supernatant (soluble fraction) were analysed by SDS-PAGE and Coomassie staining for the presence of the respective protein fusion.

Growth curves. The plasmids pMALp2x-F1, pMALp2x-F2, pMALp2x-F3, pMALc2x-F1, pMALc2x-F2 and pMALc2x-F3 were transformed into *E. coli* XL1-blue cells for protein expression. Cells were grown at 37 °C to OD_{600} 0.6 and expression was subsequently induced using 0.5 mM IPTG (Sigma) for 22 h at 25 °C. The growth rate of *E. coli* harbouring pMALp2x or pMALc2x was used as a positive control.
