# Supplementary material for: Structure–function analysis of HsiF, a gp25-like component of the type VI secretion system, in Pseudomonas aeruginosa
Source: Microbiology (Reading). 2011 Dec;157(Pt 12):3292–305. doi: 10.1099/mic.0.051987-0 (PMC3352280; doi:10.1099/mic.0.051987-0)
Supplement: Supplementary material [file supp_157.12.3292_mic051987_suppl_tables_S1-S3.pdf]

Structure–function analysis of HsiF, a gp25-like component of the type VI secretion system, in *Pseudomonas aeruginosa*

By: Nadine S. Lossi, Rana Dajani, Paul Freemont and Alain Filloux

SUPPLEMENTARY TABLES

Supplementary Table S1. Strains used in this study

Strain	Relevant characteristics	Source or reference
***E. coli* strains**		
Rosetta 2 BL21(DE3)	F^{-} *ompT* *hsdS*$_B$(r_B^{-} m_B^{-}) *gal dcm* BL21(DE3) pRARE2 (Cam^R)	Stratagene
One-shot® TOP10	F- *mcr*A Δ(*mrr-hsd*RMS-*mcr*BC) ϕ80*lac*ZΔM15 Δ*lac*X74 *rec*A1 *ara*D139 Δ(*ara-leu*) 7697 *gal*U *gal*K *rps*L (StrR) *end*A1 *nup*G	Invitrogen
XL1 blue	*recA1 endA1 gyrA96 thi-1 hsdR17 supE44 relA1 lac* [F′ *proAB lacI*qZΔM15 Tn*10* (Tetr)]	Stratagene
CC118 λpir	Host strain for pKNG101 replication; Δ(*ara-leu*) *araD* Δ*lacX74 galE galK-phoA20 thi-1 rpsE rpoB argE* (Am) *recA1* Rf^r *(lpir)*	Herrero *et al.* (1990)
***P. aeruginosa* strains**		
PAKΔ*retS*	*P. aeruginosa* PAK with a *retS* deletion	Goodman *et al.* (2004)
PAKΔ*retS*Δ*hsiF1*	*hsiF1*(PA0087) deletion mutant in PAKΔ*retS* background	This study
PAKΔ*retS*Δ*clpV1*	*clpV1* deletion mutant in PAKΔ*retS* background	Hachani *et al.* (2011)

Supplementary Table S2. Plasmids used in this study

Plasmid	Resistance*	Description	Source or reference
pCR2.1	Amp^R, Km^R	Cloning vector allowing blue-white screening (lacZα), pUC origin, Plac promoter	Invitrogen
pRK2013	Km^R	Tra^+Mob^+, helper strain for mobilization of non-self-transmissible plasmids	Figurski & Helinski (1979)
pKNG101	Str^R	*ori* R6K, *mob* RK2, *strAB sacBR*	Kaniga *et al.* (1991)
pKNG-Δ*hsiF1*	Str^R	pKNG101 containing mutator fragment for deletion of *hsiF1* (PA0087)	This study
pGEX4T1	Ap^R	Expression vector with *tac* promoter and thrombin cleavage site, N-terminal glutathione *S*-transferase (GST) fusion	GE healthcare
pGEX-F3	Ap^R	pGEX4T1 expressing HsiF3 with N-terminal GST fusion	This study
pET28a	Km^R	Expression vector with T*7* promoter; N-terminal His fusion, optional C-terminal His fusion	Novagen
pET-F1	Km^R	pET28a expressing HsiF1 with N-terminal His tag	This study
pBBR1MCS-4	Ap^R	Broad-host-range vector pBBR1 replicon	Kovach *et al.* (1995)
pHsiF1	Ap^R	pBBR1MCS-4 expressing HsiF1 with C-terminal 6×His	This study
$pHsiF1_{E105A}$	Ap^R	pBBR1MCS-4 expressing HsiF1(E105A) with C-terminal 6×His	This study
pET-F2	Km^R	pET28a expressing HsiF2 with N-terminal His fusion; primers OAL95/97	This work
pET-F3	Km^R	pET28a expressing HsiF3 with N-terminal His fusion; primers OAL98/100	This work
pGEX-F1	Ap^R	pGEX4T1 expressing HsiF1 with N-terminal GST fusion; primers OAL93/94	This work
pGEX-F2	Ap^R	pGEX4T1 expressing HsiF2 with N-terminal GST fusion; primers OAL96/97	This work
pPHO7	Ap^R	Plasmid pTZ18R containing *phoA* gene encoding alkaline phosphatase lacking its N-terminal signal peptide	Gutierrez & Devedjian (1989)
pBBR1MCS-5	Gm^R	Broad-host-range vector pBBR1 replicon	Kovach *et al.* (1995)
pHsiF1::phoA	Gm^R	pBBR1MCS-5 containing fusion of *hsiB1* to *phoA* extracted from pPHO7	This work
pHcp1::phoA	Gm^R	pBBR1MCS-5 containing fusion of *hsiB1* to *phoA* extracted from pPHO7	This work
pDsbA::phoA	Gm^R	pBBR1MCS-5 containing fusion of *hsiB1* to *phoA* gene extracted from pPHO7	This work
pMALp2x		Expression vector, fusion of gene of interest to *malE* gene directing maltose-binding protein (MBP) fusion protein to the periplasm	New England Biolabs
pMALp2x-F1		Expression vector for periplasmic expression of MBP–HsiF1	This work
pMALp2x-F2		Expression vector for periplasmic expression of MBP–HsiF2	This work

Plasmid	Resistance*	Description	Source or reference
pMAL-p2x-F3		Expression vector for periplasmic expression of MBP–HsiF3	This work
pMALc2x		Expression vector, fusion of gene of interest to *malE* gene, deleted of its signal sequence, directing MBP fusion protein to the cytosol	New England Biolabs
pMALc2x-F1		Expression vector for cytosolic expression of MBP–HsiF1	This work
pMALc2x-F2		Expression vector for cytosolic expression of MBP–HsiF2	This work
pMALc2x-F3		Expression vector for cytosolic expression of MBP–HsiF3	This work

*Ap^R, ampicillin resistance; Str^R, streptomycin resistance; Km^R, kanamycin resistance; Gm^R, gentamicin resistance.

Supplementary Table S3. Oligonucleotides used in this study

Oligonucleotide	Oligonucleotide sequence (5′→3′)
OAL65	CGGCGCTGCCGATGGTACAGACCTACT
OAL66	TCATGTACGTTCGGCCATGTCAGGCGT
OAL67	ATGGCCGAACGTACATGAACCCGCGCC
OAL68	GTAGAGCGGCAGGGCGTCCAGCGACAG
OAL87	GATGATCGCCGAAGAACTGTTG
OAL88	GAGAACTCGACGAACAGGAAGC
OAL92	CGAATTCCATATGGCCGAACTGACC
OAL94	CCGCTCGAGTCATGTACGCCTCCGCTC
OAL98	GCGCGGATCCATGTCCGGAGCCCTG
OAL100	GCGCTCGAGTCAATCGAATCGCTCATG
OAL138	GGAATTCAGGAAACAGCTATGGCCGAACTGACCCTC
OAL139	CGGGATCCTCAGTGGTGGTGATGATGATGTGTACGCCTCCGCTCGCC
OAL147	ATCGCCACCTTCGCGCCGCGCATCATCCGTTCTCT
OAL148	GATGATGCGCGGCGCGAAGGTGGCGATGGTCTCGG
OAL93	CGCGGATCCATGGCCGAACTGACC
OAL95	CGAATTCCATATGACTGGATACGGC
OAL96	CGCGGATCCATGACTGGATACGGC
OAL97	GCGCTCGAGTTAGCTGACCTTCACCTG
OAL99	CGAATTCCATATGTCCGGAGCCCTGC
OAL 748	CCCAAGCTTATAGCGTAGTGGCGCGAG
OAL 891	TCCCCCGGGTGGCCTGCACGTTCTGGCG
OAL 878	CCCAAGCTTGAGCGGCACTGACCTCGC
OAL 888	TCCCCCGGGTCTTCTTGGCCGCTGCGCG
OAL 890	TCCCCCGGGTTGTACGCCTCCGCTCGCC
OAL 746	CCCAAGCTTTGCGTGAACTGGACTTCG

Supplementary references

Figurski, D. H. & Helinski, D. R. (1979). Replication of an origin-containing derivative of plasmid RK2 dependent on a plasmid function provided in trans. *Proc Natl Acad Sci U S A* 76, 1648–1652. doi:10.1073/pnas.76.4.1648

Medline

Goodman, A. L., Kulasekara, B., Rietsch, A., Boyd, D., Smith, R. S. & Lory, S. (2004). A signaling network reciprocally regulates genes associated with acute infection and chronic persistence in *Pseudomonas aeruginosa*. *Dev Cell* **7**, 745–754. doi:10.1016/j.devcel.2004.08.020

Medline

Gutierrez, C. & Devedjian, J. C. (1989). A plasmid facilitating in vitro construction of phoA gene fusions in Escherichia coli. *Nucleic Acids Res* **17**, 3999. doi:10.1093/nar/17.10.3999

Medline

Hachani, A., Lossi, N. S., Hamilton, A., Jones, C., Bleves, S., Albesa-Jové, D. & Filloux, A. (2011). Type VI secretion system in *Pseudomonas aeruginosa*: secretion and multimerization of VgrG proteins. *J Biol Chem* **286**, 12317–12327. doi:10.1074/jbc.M110.193045

Medline

Herrero, M., de Lorenzo, V. & Timmis, K. N. (1990). Transposon vectors containing non-antibiotic resistance selection markers for cloning and stable chromosomal insertion of foreign genes in gram-negative bacteria. *J Bacteriol* **172**, 6557–6567. Medline

Kaniga, K., Delor, I. & Cornelis, G. R. (1991). A wide-host-range suicide vector for improving reverse genetics in gram-negative bacteria: inactivation of the blaA gene of *Yersinia enterocolitica*. *Gene* **109**, 137–141. doi:10.1016/0378-1119(91)90599-7

Medline

Kovach, M. E., Elzer, P. H., Hill, D. S., Robertson, G. T., Farris, M. A., Roop, R. M., II & Peterson, K. M. (1995). Four new derivatives of the broad-host-range cloning vector pBBR1MCS, carrying different antibiotic-resistance cassettes. *Gene* **166**, 175–176. doi:10.1016/0378-1119(95)00584-1 (for S data).

Medline
